# Transmission thresholds for the spread of infections in healthcare facilities

**DOI:** 10.1371/journal.pcbi.1013577

**Published:** 2025-10-15

**Authors:** Damon J. A. Toth, Karim Khader, Christopher Mitchell, Matthew H. Samore

**Affiliations:** 1 Division of Epidemiology, Department of Internal Medicine, University of Utah School of Medicine, Salt Lake City, Utah, United States of America; 2 Department of Veterans Affairs Salt Lake City Healthcare System, Salt Lake City, Utah, United States of America; 3 Department of Mathematics, University of Utah, Salt Lake City, Utah, United States of America; 4 Department of Quantitative and Qualitative Health Sciences, University of Texas School of Public Health San Antonio, San Antonio, Texas, United States of America; Stockholms Universitet, SWEDEN

## Abstract

Some infections may be sustained in the human population by persistent transmission among patients in healthcare facilities, including patients colonized with multi-drug-resistant organisms posing a major health threat. A nuanced understanding of facility characteristics that contribute to crossing a threshold for self-sustaining outbreak potential may be crucial to designing efficient interventions for lowering regional disease burden and preventing high-risk infections. Using a mathematical model, we define the facility basic reproduction number *R*_0_, where a single facility can sustain an outbreak without ongoing importation under the threshold condition *R*_0_ > 1. We define *R*_0_ for a general model with heterogeneous patient susceptibility and transmissibility and with generic length-of-stay assumptions, and we provide a software package for numerical calculation of user-defined examples. We estimate *R*_0_ using published data for carbapenemase–producing Enterobacteriaceae (CPE) in long-term acute-care hospitals (LTACHs) and the effects of interventions on *R*_0_, including surveillance, pathogen reduction treatments, and length-of-stay reduction. In a simple model, *R*_0_ is directly proportional to the sum of the mean and variance-to-mean ratio of the length-of-stay distribution. In intervention models, *R*_0_ depends on the moment-generating function of the length-of-stay distribution. From the CPE data, we estimated *R*_0_ = 1.24 (95% CI: 1.04, 1.45) prior to intervention. Weekly surveillance with 50% transmission reduction of detected patients alone could have reduced *R*_0_ to 0.85 (0.72, 0.98), with additional reduction if detected patients could be decolonized. Reducing the mean length of stay does not necessarily reduce *R*_0_ if the variance-to-mean ratio is not also reduced. We conclude that *R*_0_ > 1 conditions plausibly exist in LTACHs, where CPE outbreaks could be sustained by patients who acquire colonization and subsequently transmit to other patients during the same hospital stay. Our findings illuminate epidemiological mechanisms producing those conditions and their relationship to interventions that could efficiently improve population health.

## Author summary

The spread of infectious diseases between patients admitted to the same healthcare facility can cause large outbreaks of dangerous organisms such as bacteria resistant to available antibiotic treatments. One way to quantify facility transmission is with the basic reproduction number, $R_{\text{0}}$. When a facility patient acquires the organism, $R_{\text{0}}$ is the average number of onward transmissions from that patient, when all other patients are susceptible. There is an important threshold effect at $R_{\text{0}}=\text{1}$: when $R_{\text{0}}<\text{1}$, a single introduction is unlikely to set off a long-lasting outbreak, but if $R_{\text{0}}>\text{1}$, a continual chain of transmissions stemming from one introduction is possible. We provide a novel formula for $R_{\text{0}}$ specific to healthcare facilities and exhibiting this threshold effect. We show that $R_{\text{0}}>\text{1}$ likely occurred in facilities with high rates of drug-resistant bacterial infections, and we provide nuanced insights into intervention efforts to push $R_{\text{0}}<\text{1}$ in those facilities. Our formulas reveal that $R_{\text{0}}$ depends on statistical properties of healthcare facilities that are underappreciated for their epidemiological importance, such as the variance of patient stay duration. Our publicly available software allows other researchers to analyze models with customizable facility and disease properties and design intervention strategies for efficient reduction of facility outbreak risk.

## 1 Introduction

Infections caused by multi-drug resistant organisms (MDROs) have limited available treatments and pose a significant health threat [1]. Some MDROs have shown the ability to disseminate rapidly among patients within and between certain healthcare facilities [2], suggesting that efforts to reduce patient-to-patient transmission via inter-facility coordination [3] and/or interventions targeted at high-risk settings [4,5] could provide substantial population benefit. Results from simulations of regional outbreaks and interventions suggest that, in some scenarios, efforts to prevent transmission such as active surveillance for MDRO carriage among high-risk patients could drastically reduce regional vulnerability to explosive outbreaks that would lead to endemic presence of the MDRO [4]. These results point to the possibility that rates of MDRO transmission between healthcare patients could be close to the threshold required to sustain long-lasting chains of person-to-person transmission that are characteristic of successful invasion of a novel transmissible organism in a population.

The quantitative theory of transmission thresholds has generated a rich body of research. Levels of transmission for an organism in a population relative to a threshold are often characterized by the basic reproduction number, $R_{\text{0}}$, the expected total number of transmissions from a typical infected individual in an unexposed population [6]. If $R_{\text{0}}$ is larger than the threshold of 1, the organism can invade and produce a sustained outbreak over many generations of transmission. When above the threshold, the value of $R_{\text{0}}$ can determine the theoretical level of transmission-reducing intervention effort, such as vaccination, required to control outbreaks [7].

Some studies have applied transmission threshold and reproduction number theory specifically to organisms transmitted primarily in healthcare settings, usually defining $R_{\text{0}}$ as the expected number of transmissions from an admitted carrier of an organism during a single stay in a healthcare facility under specific assumptions for a particular pathogen [8–11]. Others have defined this quantity as the admission reproduction number, $R_{A}$ [12–17], or ward reproduction ratio, $R_{w}$ [18], noting that the quantity is less than $R_{\text{0}}$ when a single facility or ward stay may represent only a portion of the patient’s potential to transmit during the infectious period.

While the reproduction number in a healthcare facility is partly determined by properties of the organism and its typical course of colonization and infection in humans, it can also be affected by actions the facility takes to prevent patient-to-patient transmission. Of particular interest are scenarios where a facility’s $R_{\text{0}}>\text{1}$ for an MDRO under the facility’s standard transmission control efforts, but additional actions, such as active surveillance and contact precautions for detected carriers of an MDRO [19] or decolonization via pathogen reduction approaches [5,20], could potentially move $R_{\text{0}}$ below threshold.

Our aims for this work were first to define $R_{\text{0}}$ for a broad class of mathematical models of a single healthcare facility and demonstrate its utility as a threshold for facility outbreak potential, and second to demonstrate our model’s utility in assessing intervention components that might push a vulnerable facility below threshold. For this second aim, we use data from a bundled intervention that successfully reduced infections with carbapenemase-producing Enterbacteriaceae (CPE) in long-term acute care hospitals (LTACHs) [21]. Due to their composition of acutely ill patients with long length of stay, LTACHs are high-risk settings for infections with CPE and other high-priority organisms [22]. Efforts to interrupt transmission could provide substantial benefit to LTACHs and other facilities linked by patient exchange [4]. Therefore, understanding the role of individual intervention components for reducing transmission could be crucial to achieving maximum efficiency in regional outbreak prevention.

The overall outline of our study is as follows. First, we formulate differential equations describing patient states in a healthcare facility pertaining to colonization with and susceptibility to an infectious organism as well as admission and discharge. Next, we derive formulas for $R_{\text{0}}$, the expected transmissions from a patient who acquires colonization during a facility stay, in an otherwise fully susceptible facility population and with no ongoing importation. We also derive a formula for the equilibrium facility patient state distribution when the admission state distribution (including the importation rate of colonized patients) is constant. We use this equilibrium equation to estimate model parameters from CPE data from LTACHs in which constant importation and steady state facility prevalence were observed, and we estimate $R_{\text{0}}$ using those parameter estimates and the formulas we had derived. Finally, we investigate nuances on the effects of interventions on reducing $R_{\text{0}}$, to provide insights on how similar LTACHs without ongoing CPE importation could substantially reduce invasion risk by keeping $R_{\text{0}}<\text{1}$.

## 2 Methods

### 2.1 General model description

First, we defined a general class of mathematical models describing states of patients in a healthcare facility. We split the patients into different states enumerated by integer subscripts i, and let $x_{i}(t)$ be the probability that a patient is alive, not discharged, and in state i at time t after admission. Defining the column vector of probabilities 𝐱(t) containing each $x_{i}(t)$, we define the system of differential equations


$$\frac{d𝐱}{dt}=𝐖𝐱-h(t)𝐱,\;\;𝐱(\text{0})={𝐱}_{\text{a}}.$$ (1)

Here, 𝐖 contains state-to-state transition rates and may also contain constant, state-specific facility removal rates by death or live discharge. The hazard function h(t) is a facility removal rate at time t of the stay, where the same function applies to every state. The state-specific removal rates and/or the hazard function can be used to calibrate a model to an overall observed patient length of stay distribution. The vector ${𝐱}_{\text{a}}$ is the distribution of states at admission. For this system form, we derive the formula for the equilibrium cross-sectional state distribution of facility patients, under the assumption that patients are continually admitted to the facility at a constant rate with an unchanging admission state probability distribution.

Next, for modeling patient states pertaining to a particular organism that can colonize or infect patients in the facility, we assume the patient states 𝐱 are comprised of n susceptible states $\left(S_{\text{1}},\ldots ,S_{n}\right)$ and m colonized states $\left(C_{\text{1}},\ldots ,C_{m}\right)$. The colonized states represent patients carrying the modeled organism. The m different colonized states could represent any epidemiologically relevant differences between different groups of colonized patients, for example, states of infection at different body sites, different levels of transmissibility to other patients, different hazards of death or discharge, etc. The susceptible states represent patients not colonized by the organism. The n different susceptible states could be distinguished by different risks of acquiring colonization per level of exposure and/or by other relevant differences.

We use the following notation:


$$𝐱=(\begin{array}{c} S_{\text{1}}\\ \vdots \\ S_{n}\\ C_{\text{1}}\\ \vdots \\ C_{m} \end{array}),\;\;{𝐱}_{\text{a}}=(\begin{array}{c} \left(\text{1}-p_{\text{a}}\right)\theta_{\text{1}}\\ \vdots \\ \left(\text{1}-p_{\text{a}}\right)\theta_{n}\\ p_{\text{a}}\kappa_{\text{1}}\\ \vdots \\ p_{\text{a}}\kappa_{m} \end{array}),$$ (2)

$$𝐖=(\begin{array}{cccccc} -s_{\text{1}\text{1}}-s_{\text{1}}\alpha -\omega_{\text{1}} & \cdots & s_{\text{1}n} & r_{\text{1}\text{1}} & \cdots & r_{\text{1}m}\\ \vdots & \ddots & \vdots & \vdots & \ddots & \vdots \\ s_{n\text{1}} & \cdots & -s_{nn}-s_{n}\alpha -\omega_{n} & r_{n\text{1}} & \cdots & r_{nm}\\ a_{\text{1}\text{1}}\alpha & \cdots & a_{\text{1}n}\alpha & -c_{\text{1}\text{1}}-\omega_{n+\text{1}} & \cdots & c_{\text{1}m}\\ \vdots & \ddots & \vdots & \vdots & \ddots & \vdots \\ a_{m\text{1}}\alpha & \cdots & a_{mn}\alpha & c_{m\text{1}} & \cdots & -c_{mm}-\omega_{n+m} \end{array})$$ (3)

In the initial state vector ${𝐱}_{\text{a}}$ describing the admission state distribution, $p_{\text{a}}$ is the overall probability of being in a colonized state at admission, $\left(\theta_{\text{1}},\ldots ,\theta_{n}\right)$ is the admission state distribution among the susceptible state admissions, with $\sum \theta_{i}=\text{1}$, and $\left(\kappa_{\text{1}},\ldots \kappa_{m}\right)$ is the admission state distribution among the colonized state admissions, with $\sum \kappa_{i}=\text{1}$.

In the matrix 𝐖, the terms $\left(\omega_{\text{1}},\ldots ,\omega_{n+m}\right)$ in the diagonal elements are the rates of removal from the facility for patients in each state, and the other terms describe transitions from one state to another. The upper-left n×n portion of 𝐖 contains elements describing the transitions out of and between the susceptible states. The $s_{ij}$ terms are the rates of transitioning from one susceptible state to another, with $s_{jj}=\sum_{i\neq j}s_{ij}$ if n>1 and $s_{\text{1}\text{1}}=\text{0}$ if n=1. The $s_{j}\alpha$ terms are the rates of transitioning from each susceptible state to a colonized state (rates of acquisition). The $s_{j}$ coefficients describe relative susceptibility to acquisition of patients in each susceptible state, and α is a baseline acquisition rate that is assumed to depend on the prevalence of patients in the colonized states, who can transmit to the susceptible patients, as follows:


$$\alpha =\beta_{\text{1}}C_{\text{1}}+\cdots +\beta_{m}C_{m},$$ (4)

where the $\left(\beta_{\text{1}},\ldots ,\beta_{m}\right)$ coefficients define the relative transmissibility of patients in the different colonized states.

The lower-left m×n portion of 𝐖 contains rates of transitioning into each colonized state from each susceptible state, with $s_{j}=\sum a_{ij}$. The lower-right m×m portion of 𝐖 contains elements describing the transitions out of and between the colonized states. For the $c_{jj}$ elements in the diagonal we have $c_{jj}=\sum_{i\neq j}c_{ij}+\sum r_{ij}$, where the $r_{ij}$ terms comprising the upper right n×m portion of 𝐖 are the rates of transitioning from colonized to susceptible, i.e., the rates of clearing colonization.

For this system, we derive a formula for the facility basic reproduction number $R_{\text{0}}$ for given values of the importation state distribution, state transition rates, and relative transmissibility parameters defined above. We derive our equation to match the “basic reproduction ratio” formula in Diekmann et al [6]. Their definition depends on quantifying an individual’s state just prior to acquiring colonization and the expected infectivity of an individual who was in a given state just prior to acquisition at a given prior time, toward a susceptible individual currently in a given state. For our model, the state of a facility patient is defined as the susceptible compartment the patient is in and the time since admission. Hence the $R_{\text{0}}$ formula involves both summation over discrete patient states and continuous-time integration of functions involving the time since admission. Details are provided in S1 Text.

We also derive formulas for the equilibrium cross-sectional patient state distribution when the admission rate of patients in each state is constant, as described further in subsequent sections. The model is not designed to represent transient dynamics of a facility patient population starting from a non-equilibrium state distribution. In full generality, such a model requires representing patient states as a multivariate function 𝐱(τ,t) that tracks the number of facility patients in each state who were admitted τ time units ago, at time t after the start of the dynamic simulation. While studying transient dynamics is outside the scope of this study, we provide an example of a model formulation of this type in S1 Text.

### 2.2 Example model descriptions

We use our general formula for $R_{\text{0}}$ to derive $R_{\text{0}}$ formulae for a series of specific model examples.

#### 2.2.1 Model 1: Simple susceptible–colonized model.

We specified a simple version of our general disease model with only two states and one direction of transition between states. The state probability vector 𝐱 consists of one susceptible state $\left(S_{\text{1}}\right)$ that we rename S and one colonized state ${(C}_{\text{1}})$ that we rename C. The admission state probability vector in the above notation contains $\theta_{\text{1}}=\text{1}$ and $\kappa_{\text{1}}=\text{1}$:


$$𝐱=(\begin{array}{c} S\\ C \end{array}),\;\;{𝐱}_{\text{a}}=(\begin{array}{c} \text{1}-p_{\text{a}}\\ p_{\text{a}} \end{array}).$$ (5)

We assume that susceptible inpatients acquire colonization at rate α, and there are no other state transitions or removals other than removal modeled by the hazard function h(t). Thus, in the general model notation, we have $s_{\text{1}}=\text{1},\;a_{\text{1}\text{1}}=\text{1},\;r_{\text{1}\text{1}}=\text{0},\omega_{\text{1}}=\omega_{\text{2}}=\text{0}$. The state transition matrix 𝐖 is then


$$𝐖=(\begin{array}{cc} -\alpha & \text{0}\\ \alpha & \text{0} \end{array}).$$ (6)

For transmission, there is only one colonized state from which transmissions can occur, so there is a single transmission parameter ($\beta_{\text{1}})$ that we rename β, and the acquisition rate α=βC. The time-of-stay-dependent removal hazard h(t) is left general. The equivalent system in non-matrix form is as follows:


$$\frac{dS}{dt}=-\left(\beta C+h(t)\right)S,\;\;S(\text{0})=\text{1}-p_{\text{a}}$$ (7)

$$\frac{dC}{dt}=\beta CS-h(t)C,\;\;C(\text{0})=p_{\text{a}}$$ (8)

#### 2.2.2 Model 2: Clearance of colonization.

To extend Model 1, we add a non-zero clearance rate γ of moving from colonized back to susceptible. In the general model notation, we have $s_{\text{1}}=\text{1},\;a_{\text{1}\text{1}}=\text{1},\;r_{\text{1}\text{1}}=\gamma ,\omega_{\text{1}}=\omega_{\text{2}}=\text{0}$. The state transmission matrix 𝐖 is then


$$𝐖=(\begin{array}{cc} -\alpha & \gamma \\ \alpha & -\gamma \end{array}).$$ (9)

In non-matrix equation form, we have:


$$\frac{dS}{dt}=-\left(\beta C+h(t)\right)S+\gamma C,\;\;S(\text{0})=\text{1}-p_{\text{a}}$$ (10)

$$\frac{dC}{dt}=\beta CS-\left(\gamma +h(t)\right)C,\;\;C(\text{0})=p_{\text{a}}$$ (11)

#### 2.2.3 Model 3: Clinical detection with contact precautions.

For this model, we extend Model 2 to include two colonized states $\left(C_{\text{1}},C_{\text{2}}\right)$ that we rename C and $C_{\text{cd}}$. These two colonized states represent, respectively, patients with no prior detection of their colonization and patients with a prior clinical detection. This type of model is generally used to represent the transition from asymptomatic colonization to the onset of symptomatic, invasive infection that triggers a clinical test for the causative organism. For the admission state probability vector in the above notation, we assume $\theta_{\text{1}}=\text{1}$, $\kappa_{\text{1}}=\text{1}$, and $\kappa_{\text{2}}=\text{0}$, i.e., all colonized patients are undetected at admission:


$$𝐱=(\begin{array}{c} S\\ C\\ C_{\text{cd}} \end{array}),\;\;{𝐱}_{\text{a}}=(\begin{array}{c} \text{1}-p_{\text{a}}\\ p_{\text{a}}\\ \text{0} \end{array}).$$ (12)

For state transitions among inpatients, we assume that susceptible patients acquiring colonization (at rate α) move into the undetected colonized state. Undetected colonized patients move to the susceptible state at clearance rate γ and move to the clinically detected state at clinical detection rate $\delta_{\text{c}}$, which represent a progression rate from asymptomatic colonization to confirmed clinical infection. Clinically detected patients are assumed to remain in that state until the end of the stay. Thus, in the general model notation, we have:


$$s_{\text{1}}=\text{1},\;a_{\text{1}\text{1}}=\text{1},\;a_{\text{2}\text{1}}=\text{0},\;r_{\text{1}\text{1}}=\gamma ,\;c_{\text{2}\text{1}}=\delta_{\text{c}},\;r_{\text{1}\text{2}}=\text{0},c_{\text{1}\text{2}}=\text{0},\omega_{\text{1}}=\omega_{\text{2}}=\omega_{\text{3}}=\text{0}$$ (13)

The state transmission matrix 𝐖 is then


$$𝐖=(\begin{array}{ccc} -\alpha & \gamma & \text{0}\\ \alpha & -\gamma -\delta_{\text{c}} & \text{0}\\ \text{0} & \delta_{\text{c}} & \text{0} \end{array})$$ (14)

For the relative transmission rates from patients in the two colonized states, we assume that clinically detected patients transmit at a rate reduced according to the effectiveness ε of contact precautions: $\beta_{\text{1}}=\beta ,\;\beta_{\text{2}}=\beta (\text{1}-\varepsilon )$. Then the acquisition rate $\alpha =\beta \left(C+(\text{1}-\varepsilon )C_{\text{cd}}\right).$

In non-matrix equation form, Model 3 is written as follows:


$$\frac{dS}{dt}=-\left(\beta \left(C+(\text{1}-\varepsilon )C_{\text{cd}}\right)+h(t)\right)S+\gamma C,\;\;S(\text{0})=\text{1}-p_{\text{a}}$$ (15)

$$\frac{dC}{dt}=\beta \left(C+(\text{1}-\varepsilon )C_{\text{cd}}\right)S-\left(\gamma -\delta_{\text{c}}+h(t)\right)C,\;\;C(\text{0})=p_{\text{a}}$$ (16)

$$\frac{dC_{\text{cd}}}{dt}=\delta_{\text{c}}C-h(t)C_{\text{cd}},\;\;C_{\text{cd}}(\text{0})=\text{0}$$ (17)

#### 2.2.4 Model 4: Active surveillance and decolonization.

We extend Model 3 to include two simultaneous interventions: active surveillance to detect patients with colonization who have not been detected by clinical test, and decolonization of those detected patients via administration of a decolonizing drug or pathogen reduction agent. Patients detected by active surveillance are assumed to be placed under contact precautions in addition to receiving the pathogen reduction treatment, which increases the rate of clearance above the natural clearance rate.

The state probability vector 𝐱 consists of two susceptible states ${(S}_{\text{1}},S_{\text{2}})$ that we rename S and $S_{\text{sd}}$ and three colonized states ${(C}_{\text{1}},C_{\text{2}},C_{\text{3}})$ that we rename $C,C_{\text{sd}}$, and $C_{\text{cd}}$. The new states with subscript “sd” represent patients with a positive surveillance test earlier in the facility stay. At admission, we assume that susceptible patients never test positive, and colonized patients test positive with probability $\pi_{a}$, which incorporates the probability of receiving an admission surveillance test and the probability that the test avoids a false negative result. For the admission state probability vector in the above notation, we assume $\theta_{\text{1}}=\text{1}$, $\theta_{\text{2}}=\text{0}$, $\kappa_{\text{1}}=\text{1}-\pi_{a}$, $\kappa_{\text{2}}=\pi_{a}$, $\kappa_{\text{3}}=\text{0}$:


$$𝐱=(\begin{array}{c} S\\ S_{\text{sd}}\\ C\\ C_{\text{sd}}\\ C_{\text{cd}} \end{array}),\;\;{𝐱}_{\text{a}}=(\begin{array}{c} \text{1}-p_{\text{a}}\\ \text{0}\\ \left(\text{1}-\pi_{a}\right)p_{\text{a}}\\ \pi_{a}p_{\text{a}}\\ \text{0} \end{array}).$$ (18)

For state transitions among inpatients, we assume that undetected colonized patients become surveillance detected at rate $\delta_{\text{s}}$, representing mid-stay surveillance performed on patients who did not test positive at admission. Surveillance-detected, colonized patients can move to the clinically detected state at the same rate $\delta_{\text{c}}$ as undetected colonized patients. The new susceptible state $S_{\text{sd}}$ can be reached by surveillance-detected, colonized patients who clear colonization during the stay. Surveillance-positive patients are assumed to remain under contact precautions for their entire remaining stay. For susceptible patients under contact precautions, we assume that those precautions reduced their rate of re-acquisition by the same factor, (1−ε), that they reduce transmissibility. In the general model notation we have the following nonzero elements: $s_{\text{1}}=a_{\text{1}\text{1}}=\text{1}$, $s_{\text{2}}=a_{\text{2}\text{2}}=\text{1}-\varepsilon$, $r_{\text{1}\text{1}}=\gamma$, $c_{\text{2}\text{1}}=\delta_{\text{s}}$, $c_{\text{3}\text{1}}=\delta_{\text{c}}$, $r_{\text{3}\text{2}}=\gamma_{\text{d}}$, $c_{\text{3}\text{2}}=\delta_{\text{c}}$, $\omega_{\text{1}}=\omega_{\text{2}}=\omega_{\text{3}}=\omega_{\text{4}}=\omega_{\text{5}}=\text{0}$. The state transmission matrix 𝐖 is then


$$𝐖=(\begin{array}{ccccc} -\alpha & \text{0} & \gamma & \text{0} & \text{0}\\ \text{0} & -(\text{1}-\varepsilon )\alpha & \text{0} & \gamma_{\text{d}} & \text{0}\\ \alpha & \text{0} & -\gamma -\delta_{\text{s}}-\delta_{\text{c}} & \text{0} & \text{0}\\ \text{0} & (\text{1}-\varepsilon )\alpha & \delta_{\text{s}} & -\gamma_{\text{d}}-\delta_{\text{c}} & \text{0}\\ \text{0} & \text{0} & \delta_{\text{c}} & \delta_{\text{c}} & \text{0} \end{array})$$ (19)

For relative transmission rates, we assume that surveillance detected, colonized patients transmit at a rate commensurate with clinically detected patients: $\beta_{\text{1}}=\beta ,\;\beta_{\text{2}}=\beta_{\text{3}}=\beta (\text{1}-\varepsilon )$. Then the acquisition rate $\beta_{\text{1}}=\beta ,\;\beta_{\text{2}}=\beta_{\text{3}}=\beta (\text{1}-\varepsilon )$.

In non-matrix equation form, Model 4 is written as follows:


$$\frac{dS}{dt}=-\left(\beta \left(C+(\text{1}-\varepsilon )\left(C_{\text{cd}}+C_{\text{sd}}\right)\right)+h(t)\right)S+\gamma C,\;\;S(\text{0})=\text{1}-p_{\text{a}}$$ (20)

$$\frac{dS_{\text{sd}}}{dt}=-\left((\text{1}-\varepsilon )\beta \left(C+(\text{1}-\varepsilon )\left(C_{\text{cd}}+C_{\text{sd}}\right)\right)+h(t)\right)S_{\text{sd}}+\gamma_{\text{d}}C_{\text{sd}},\;\;S_{\text{sd}}(\text{0})=\text{0}$$ (21)

$$\frac{dC}{dt}=\beta \left(C+(\text{1}-\varepsilon )\left(C_{\text{cd}}+C_{\text{sd}}\right)\right)S-\left(\gamma -\delta_{\text{s}}-\delta_{\text{c}}+h(t)\right)C,\;\;C(\text{0})=\left(\text{1}-\pi_{a}\right)p_{\text{a}}$$ (22)

$$\frac{dC_{\text{sd}}}{dt}=(\text{1}-\varepsilon )\beta \left(C+(\text{1}-\varepsilon )\left(C_{\text{cd}}+C_{\text{sd}}\right)\right)S_{\text{sd}}+\delta_{\text{s}}C-\left(\gamma_{\text{d}}-\delta_{\text{c}}+h(t)\right)C_{\text{sd}},\;\;C_{\text{sd}}(\text{0})=\pi_{a}p_{\text{a}}$$ (23)

$$\frac{dC_{\text{cd}}}{dt}=\delta_{\text{c}}C+\delta_{\text{c}}C_{\text{sd}}-h(t)C_{\text{cd}},\;\;C_{\text{cd}}(\text{0})=\text{0}$$ (24)

### 2.3 Calibration of Model 3 to pre-intervention CPE in LTACH

As in our prior work [5,23], we calibrated model parameter values to data for CPE in Chicago-area LTACHs [21]. Prior to intervention in those LTACHs, cross-sectional prevalence surveys revealed nearly 50% prevalence of CPE carriers, and there were significant rates of CPE clinical detection (Table 1). The facilities did not routinely perform CPE surveillance before the intervention, thus we used our Model 3 to represent the pre-intervention scenario with no active surveillance. For the facility removal (discharge or death) hazard, we used a function giving rise to a length-of-stay distribution governed by a mixture of an exponential and a gamma distribution, which in prior work we found to provide a parsimonious fit to detailed LTACH length of stay data [4]. The distribution has a total of four parameters: the rate parameter of an exponential distribution, $r_{x}$, the rate and shape parameters of a gamma distribution, $r_{g}$ and k, and the portion of patients following the exponential distribution, $p_{x}$. We calculated the unique values of these four parameters that produce a length-of-stay distribution matching the following statistics reported in the LTACH study [21]: the mean, median, and interquartile range of hospital stay (S1 Text).

**Table 1:** Data from pre-intervention LTACHs [21].

Value (symbol)	Data	95% range
Admission CPE positivity fraction (a)	0.206	(0.189, 0.223)
Cross-sectional facility CPE positivity fraction (f)	0.458	(0.421, 0.495)
CPE clinical detection rate per 1000 patient days (d)	3.7	(3.4, 4.0)
Days of stay: mean; 25^th^, 50^th^, 75^th^ percentiles (μ, $l_{\text{2}\text{5}}$, $l_{\text{5}\text{0}}$, $l_{\text{7}\text{5}}$)	33.8; 16, 28, 43	–

For the CPE parameters in Model 3, we assumed the CPE clearance rate γ=1/ (387 days) [24], contact precaution effectiveness ε=0.5 [25], and CPE test sensitivity = 0.85 [26,27]. Because the LTACH importation rate and cross-sectional prevalence of CPE were observed and stable over time, we assumed the Model 3 system was at equilibrium, which means that the acquisition rate, α, was constant, making Model 3 a linear system. We solved for the value of α and of the progression rate to clinical detection, $\delta_{\text{c}}$, that produced a match to the equilibrium cross-sectional CPE carriage prevalence (scaled by the assumed test sensitivity) and CPE clinical detection incidence reported in the source LTACH study (Table 1). Then we calculated the baseline transmission rate, β, that produced the correct acquisition rate under the calibrated model (Table 2 and S1 Text). Finally, we used the assumed values of γ and ε and the data-calibrated values of β and $\delta_{\text{c}}$ to calculate $R_{\text{0}}$ using the formula we derived for Model 3 (see Results).

**Table 2:** Model 3 parameters and assumptions.

Parameter	Assumed value or formula
Clearance rate of CPE carriers per day (γ)	1/ 387
Contact precaution effectiveness (ε)	0.5
Surveillance test sensitivity (σ)	0.85
CPE importation rate ($p_{\text{a}}$)	a/σ
Length of stay parameters ($p_{x}$,$r_{x}$, $r_{g}$, k)	see results
CPE baseline per-capita transmission rate per day (β)	see results
CPE clinical detection rate of CPE carriers ($\delta_{\text{c}})$	see results

The value of a in the importation rate formula is the fraction of patients positive by surveillance test at admission (Table 1). The values of the length of stay parameters were calibrated to match the observed length of stay statistics (Table 1), and the values of β and $\delta_{\text{c}}$ were calibrated to produce a model equilibrium matching the cross-sectional facility CPE positivity fraction and clinical detection rate data (Table 1).

We accounted for uncertainty arising from certain pre-intervention data that were used for calibration (admission CPE positivity and surveillance adherence fractions), or calibration targets (cross-sectional CPE positivity fraction and clinical detection incidence) in the model. Here, we relied on the reported uncertainty ranges from the source paper [21] (Table 1). Each value with reported uncertainty was derived from a large number of observations [21], justifying the use of a normal distribution approximation to the underlying distribution. We incorporated these assumed parameter distributions into a Latin hypercube sampling scheme [28], which produces random sets of values representative of the multi-variate distribution. We used 1000 combinations of the paper-derived values from the Latin hypercube sampling scheme to create 95% intervals for our results, including $R_{\text{0}}$.

### 2.4 Application of Model 4 to intervention analysis for CPE in LTACH

We used Model 4 to investigate the potential effects of active surveillance and decolonization for CPE carriage on reducing the facility reproduction number from the pre-intervention estimates. First, we assessed the biweekly surveillance component of the intervention done in the source study [21], which in our prior work we found could largely explain the post-intervention CPE reduction they observed when 50% contact precaution effectiveness was assumed [23]. The surveillance detection rate, $\delta_{\text{s}}$, was assumed to be the product of the targeted testing rate (once per 14 days), the reported compliance rate (0.954), and the assumed test sensitivity (0.85): $\delta_{\text{s}}\approx \;\text{0}.\text{058}$ per day. We tested the effect of increasing the frequency of surveillance from biweekly to weekly and calculated the minimum frequency of surveillance testing required to make $R_{\text{0}}<\;\text{1}$.

Next, we modeled the effect of combining surveillance with pathogen reduction treatment of surveillance-detected patients, represented by increasing the non-treated rate of clearing colonization among surveillance-detected patients by a factor f> 1, such that $\gamma_{\text{d}}=f\gamma$. For values of *f* = 2, 5, 10, 50, 100, and ∞ (immediate decolonization at detection), we calculated $R_{\text{0}}$ for weekly and biweekly surveillance, and the surveillance rate required to achieve $R_{\text{0}}<\;\text{1}$.

### 2.5 Length of stay intervention analysis

We tested the effects of interventions that would alter one or more of the parameters governing the length of stay distribution on $R_{\text{0}}$, using Model 3 calibrated to LTACH CPE data (Tables 1 and 2) as the starting point. As the overall mean length of stay decreases as either of the rate parameters $r_{x}$ or $r_{g}$ increases, we tested the $R_{\text{0}}$-altering effects of increasing either $r_{x}$, $r_{g}$, or both while holding the other parameters constant, to model the effects of efforts to speed up the discharge of LTACH patients. We also tested the effects of altering the $p_{x}$ parameter governing the portion of patients following exponential- or gamma-distributed length of stay in favor of the group with lower mean length of stay.

Finally, we chose random combinations of all 4 parameters governing the length-of-stay distribution and produced scatterplots of the resulting $R_{\text{0}}$ (again using Model 3 calibrated to LTACH CPE data) vs. different length-of-stay distribution statistics. We plotted $R_{\text{0}}$ against the length of stay mean, standard deviation, variance-to-mean ratio (VMR), and the sum of the mean and VMR, and we calculated Pearson’s correlation coefficient for the correlation between each of those statistics and $R_{\text{0}}$.

## 3 Results

### 3.1 General facility basic reproduction number ($R_{0}$) formula

For the general model described in the Methods section, we derived an expression for the basic reproduction number among facility patients. We express the result using the following components:


$$\begin{array}{c} 𝐒=(\begin{array}{ccc} -s_{\text{1}\text{1}}-\omega_{\text{1}} & \cdots & s_{\text{1}n}\\ \vdots & \ddots & \vdots \\ s_{n\text{1}} & \cdots & -s_{nn}-\omega_{n} \end{array}),\;\;𝐂=(\begin{array}{ccc} -c_{\text{1}\text{1}}-\omega_{n+\text{1}} & \cdots & c_{\text{1}m}\\ \vdots & \ddots & \vdots \\ c_{m\text{1}} & \cdots & -c_{mm}-\omega_{n+m} \end{array}),\\ 𝐀=(\begin{array}{ccc} a_{\text{1}\text{1}} & \cdots & a_{\text{1}n}\\ \vdots & \ddots & \vdots \\ a_{m\text{1}} & \cdots & a_{mn} \end{array}),\;\;\beta =(\begin{array}{c} \beta_{\text{1}}\\ \vdots \\ \beta_{m} \end{array}),\;\;\theta =(\begin{array}{c} \theta_{\text{1}}\\ \vdots \\ \theta_{n} \end{array}),\;\;F(t)=\text{1}-e^{-\int_{\text{0}}^{t}h(\tau )d\tau } \end{array}$$ (25)

The elements in the matrices 𝐒, 𝐀, and 𝐂 were defined within the matrix **W** in the Methods section. The matrix 𝐒 describes transition rates between the susceptible states in the absence of colonized patients, and state-specific removal rates from the facility along the diagonal ($\omega_{i}$ terms). The matrix 𝐂 contains transition rates from and between the colonized states, including state specific removal rates from the facility along the diagonal ($\omega_{i}$ terms). The matrix 𝐀 contains the relative transition rates $a_{ij}$ from susceptible state j to colonized state i upon acquisition. The vector β contains the transmission rates from each colonized state. The full transition rate from susceptible state susceptible state j to colonized state i is $a_{ij}\left(\beta_{\text{1}}C_{\text{1}}+\ldots +\beta_{m}C_{m}\right)$. The vector θ contains the fractions of admitted, susceptible patients who are in each of the susceptible states at admission. The function F(t) is the cumulative distribution function of the facility length of stay distribution of patients who are removed via the hazard function h(t).

With these elements in hand, we derived the following result for the facility basic reproduction number $R_{\text{0}}$ (S1 Text):


$$R_{\text{0}}=\frac{\beta^{\text{T}}\left(\int_{\text{0}}^{\infty }\left(\text{1}-F(t)\right)e^{𝐂t}\int_{\text{0}}^{t}e^{-𝐂\tau }𝐀e^{𝐒\tau }\theta d\tau dt\right)}{1^{\text{T}}\int_{\text{0}}^{\infty }\left(\text{1}-F(t)\right)e^{𝐒t}\theta dt}$$ (26)

This formula is a dot product of the vector β, containing the transmissibility per time of each colonized compartment, with a vector of the same length that contains the expected amount of time spent in each colonized compartment between the time of acquiring colonization in the facility and discharge or death.

We provide example models below in which $R_{\text{0}}$ can be expressed symbolically, which generally occurs when the eigenvalues of matrices 𝐒 and 𝐂 can be expressed as formulas in terms of the model parameters. Otherwise, $\;R_{\text{0}}$ can be calculated numerically as follows. In the numerator of the above expression for $R_{\text{0}}$, the vector $e^{𝐂t}\int_{\text{0}}^{t}e^{-𝐂\tau }𝐀e^{𝐒\tau }\theta d\tau$ is equivalent to final m elements of the vector $e^{\overset{―}{𝐖}t}\overset{―}{\theta }$, in which


$$\overset{―}{𝐖}=(\begin{array}{cc} 𝐒 & 0_{n\times m}\\ 𝐀 & 𝐂 \end{array}),\;\;\overset{―}{\theta }=(\begin{array}{c} \theta \\ 0_{m\times \text{1}} \end{array}),$$ (27)

and $0_{i\times j}$ contains i rows and j columns of zeros. We numerically calculate the eigenvalues $\lambda_{i}$ of $\overset{―}{𝐖}$ and associated eigenvectors to determine the linear combinations of $e^{\lambda_{i}t}$ functions that comprise the elements of $e^{\overset{―}{𝐖}t}\overset{―}{\theta }$. Then, integrating the product of this vector and (1−F(t)) produces a vector with linear combinations of $K\left(\lambda_{i}\right)$, where $K(x)=\frac{\left(\text{1}-M(x)\right)}{x}$ and M(x) is the moment generating function of the distribution for which F is the cumulative distribution function.

For a model with the hazard function h(t)=0, the patients’ rate of removal from the facility (by discharge or death) is entirely state-dependent and quantified by the $\omega_{i}$ values within the matrices. Then the function F(t)=0 and the basic reproduction number formula (S1 Text) reduces to:


$$R_{\text{0}}=-\beta^{\text{T}}{𝐂}^{-\text{1}}𝐀\left(\frac{{𝐒}^{-\text{1}}\theta }{1^{\text{T}}{𝐒}^{-\text{1}}\theta }\right)$$ (28)

This result is valid if all patients are guaranteed to eventually be removed from the facility, i.e., there is always a nonzero probability that an inpatient will eventually reach a state i from which the removal rate $\omega_{i}>\text{0}$.

We created an R package, facilityepimath [29], containing a function that numerically calculates $R_{\text{0}}$ according to the above formula, for a given set of numerical inputs for 𝐒, 𝐂, 𝐀, β, θ, and M (if h(t)≠0).

### 3.2 Example model $R_{0}$ formulas

#### 3.2.1 Model 1: Simple susceptible–colonized model.

For this model we have:


𝐒=0,  𝐂=0,  𝐀=1,  θ=1,  β=β, (29)

and the hazard function h(t) is left general. Substituting these values in the general formula gives


$$R_{\text{0}}=\frac{\beta \int_{\text{0}}^{\infty }\left(\text{1}-F(t)\right)tdt}{\int_{\text{0}}^{\infty }\left(\text{1}-F(t)\right)dt}$$ (30)

In S1 Text we show that $\int_{\text{0}}^{\infty }\left(\text{1}-F(t)\right)tdt=\frac{\left(\mu^{\text{2}}+\sigma^{\text{2}}\right)}{\text{2}}$, and $\int_{\text{0}}^{\infty }\left(\text{1}-F(t)\right)dt=\mu$, where μ and $\sigma^{\text{2}}$ are the mean and variance, respectively, of the distribution for which F(t) is the cumulative distribution function. Therefore, our result is:


$$R_{\text{0}}=\beta \left(\frac{\mu^{\text{2}}+\sigma^{\text{2}}}{\text{2}\mu }\right)$$ (31)

#### 3.2.2 Model 2: Clearance of colonization.

For this model we have:


𝐒=0,  𝐂=−γ,  𝐀=1,  θ=1,  β=β. (32)

After substitution and some integration (see more detailed steps in S1 Text), the result is:


$$R_{\text{0}}=\frac{\beta \left(\int_{\text{0}}^{\infty }\left(\text{1}-F(t)\right)dt-\int_{\text{0}}^{\infty }\left(\text{1}-F(t)\right)e^{-\gamma t}dt\right)}{\gamma \int_{\text{0}}^{\infty }\left(\text{1}-F(t)\right)dt}$$ (33)

In S1 Text demonstrate that, for a real number λ<0, $\int_{\text{0}}^{\infty }\left(\text{1}-F(t)\right)e^{\lambda t}dt=\frac{\left(M(\lambda )-\text{1}\right)}{\lambda }$, where M is the moment-generating function of the distribution for which F is the cumulative distribution function. For ease of notation, we define the function $K(\lambda )=\frac{\left(M(\lambda )-\text{1}\right)}{\lambda }$. Our result for this model can then be expressed


$$R_{\text{0}}=\frac{\beta }{\gamma }\left(\text{1}-\frac{K(-\gamma )}{\mu }\right)$$ (34)

Some commonly used parametric distributions for modeling length of stay provide convenient expressions for the moment-generating function M within the formula for K. For example, for length of stay exponentially distributed with rate r (constant removal hazard), we have $\mu =\frac{\text{1}}{r}$ and $K(\lambda )=\frac{\text{1}}{\left(r-\lambda \right)}$, which leads to:


$$R_{\text{0}}^{\text{exp}}=\frac{\beta }{r+\gamma }$$ (35)

With time to removal gamma distributed with rate parameter r and shape parameter k, we have $\mu =\frac{k}{r}$ and $K(\lambda )=\frac{\left((\text{1}-\lambda /r{)}^{-k}-\text{1}\right)}{\lambda }$, which leads to


$$R_{\text{0}}^{\text{gam}}=\frac{\beta }{\gamma }\left(\text{1}-\frac{{\text{1}-\left(\text{1}+\frac{\gamma }{r}\right)}^{-k}}{\frac{k\gamma }{r}}\right)$$ (36)

#### 3.2.3 Model 3: Clinical detection with contact precautions.

For this model we have:


$$𝐒=\text{0},\;\;𝐂=(\begin{array}{cc} -\delta_{\text{c}}-\gamma & \text{0}\\ \delta_{\text{c}} & \text{0} \end{array}),\;\;𝐀=(\begin{array}{c} \text{1}\\ \text{0} \end{array}),\;\;\theta =\text{1},\;\;\beta =(\begin{array}{c} \beta \\ \beta (\text{1}-\varepsilon ) \end{array})$$ (37)

The formula for $R_{\text{0}}$ contains $e^{𝐂t}$, which, because 𝐂 is a matrix, is the matrix exponential:


$$e^{𝐂t}=(\begin{array}{cc} e^{-\left(\delta_{\text{c}}+\gamma \right)t} & \text{0}\\ \frac{\delta_{\text{c}}}{\delta_{\text{c}}+\gamma }\left(\text{1}-e^{-\left(\delta_{\text{c}}+\gamma \right)t}\right) & \text{1} \end{array})$$ (38)

Substituting this and the other expressions in the $R_{\text{0}}$ formula, we arrive at the following results after integrating and performing matrix multiplications:


$$R_{\text{0}}=\frac{\beta }{\delta_{\text{c}}+\gamma }\left(\left(\text{1}-\frac{\delta_{\text{c}}(\text{1}-\varepsilon )}{\delta_{\text{c}}+\gamma }\right)\left(\text{1}-\frac{K\left(-\delta_{\text{c}}-\gamma \right)}{\mu }\right)+\delta_{\text{c}}(\text{1}-\varepsilon )\frac{\mu^{\text{2}}+\sigma^{\text{2}}}{\text{2}\mu }\right)$$ (39)

#### 3.2.4 Model 4: Active surveillance and decolonization.

For this model we have:


$$\begin{array}{c} 𝐒=(\begin{array}{cc} \text{0} & \text{0}\\ \text{0} & \text{0} \end{array}),\;\;𝐂=(\begin{array}{ccc} -\delta_{\text{s}}-\delta_{\text{c}}-\gamma & \text{0} & \text{0}\\ \delta_{\text{s}} & -\delta_{\text{c}}-\gamma_{\text{d}} & \text{0}\\ \delta_{\text{c}} & \delta_{\text{c}} & \text{0} \end{array}),\\ 𝐀=(\begin{array}{cc} \text{1} & \text{0}\\ \text{0} & \text{1}-\varepsilon \\ \text{0} & \text{0} \end{array}),\;\;\theta =(\begin{array}{c} \text{1}\\ \text{0} \end{array}),\;\;\beta =(\begin{array}{c} \beta \\ \beta (\text{1}-\varepsilon )\\ \beta (\text{1}-\varepsilon ) \end{array}) \end{array}$$ (40)

The matrix exponentials $e^{𝐒t}$ and $e^{𝐂t}$ can be expressed:


$$e^{𝐒t}=(\begin{array}{cc} \text{1} & \text{0}\\ \text{0} & \text{1} \end{array})$$ (41)

$$e^{𝐂t}=(\begin{array}{ccc} e^{c_{\text{1}\text{1}}t} & \text{0} & \text{0}\\ \frac{\delta_{\text{s}}}{\delta_{\text{s}}-\left(\gamma_{\text{d}}-\gamma \right)}\left(e^{c_{\text{2}\text{2}}t}-e^{c_{\text{1}\text{1}}t}\right) & e^{c_{\text{2}\text{2}}t} & \text{0}\\ \frac{\delta_{\text{c}}}{\delta_{\text{s}}-\left(\gamma_{\text{d}}-\gamma \right)}\left(\frac{\delta_{\text{s}}}{\delta_{\text{c}}+\gamma_{\text{d}}}\left(\text{1}-e^{c_{\text{2}\text{2}}t}\right)-\frac{\gamma_{\text{d}}-\gamma }{\delta_{\text{s}}+\delta_{\text{c}}+\gamma }\left(\text{1}-e^{c_{\text{1}\text{1}}t}\right)\right) & \frac{\delta_{\text{c}}}{\delta_{\text{c}}+\gamma_{\text{d}}}\left(\text{1}-e^{c_{\text{2}\text{2}}t}\right) & \text{1} \end{array})$$ (42)

The values $c_{\text{1}\text{1}}$ and $c_{\text{2}\text{2}}$ are the first and second diagonal elements of the matrix 𝐂. Substituting these and the other expressions in the $R_{\text{0}}$ formula, we arrive at the following result after integrating and performing matrix multiplications:


$$\begin{array}{cc} R_{\text{0}} & =\;\frac{\beta }{\delta_{\text{s}}+\delta_{\text{c}}+\gamma }\left(\text{1}+\frac{\text{1}-\varepsilon }{\delta_{\text{s}}-\left(\gamma_{\text{d}}-\gamma \right)}\left(\frac{\delta_{\text{c}}\left(\gamma_{\text{d}}-\gamma \right)}{\delta_{\text{s}}+\delta_{\text{c}}+\gamma }-\delta_{\text{s}}\right)\right)\left(\text{1}-\frac{K\left(-\delta_{\text{s}}-\delta_{\text{c}}-\gamma \right)}{\mu }\right)\\ & +\frac{\beta (\text{1}-\varepsilon )}{\delta_{\text{s}}-\left(\gamma_{\text{d}}-\gamma \right)}\left(\frac{\delta_{\text{s}}\gamma_{\text{d}}}{{\left(\delta_{\text{c}}+\gamma_{\text{d}}\right)}^{\text{2}}}\left(\text{1}-\frac{K\left(-\delta_{\text{c}}-\gamma_{\text{d}}\right)}{\mu }\right)+\left(\frac{\delta_{\text{c}}\delta_{\text{s}}}{\delta_{\text{c}}+\gamma_{\text{d}}}-\frac{\delta_{\text{c}}\left(\gamma_{\text{d}}-\gamma \right)}{\delta_{\text{s}}+\delta_{\text{c}}+\gamma }\right)\frac{\mu^{\text{2}}+\sigma^{\text{2}}}{\text{2}\mu }\right) \end{array}$$ (43)

In the case that $\delta_{\text{s}}-\left(\gamma_{\text{d}}-\gamma \right)=\text{0}$, the above expression is indeterminate because of division by zero; this corresponds to the matrix 𝐂 having a repeated eigenvalue ($c_{\text{1}\text{1}}=c_{\text{2}\text{2}}=\delta_{\text{s}}-\delta_{\text{c}}-\gamma$) that is degenerate, and the matrix exponential $e^{𝐂t}$ includes eigenfunctions $e^{c_{\text{1}\text{1}}t}$ and $te^{c_{\text{1}\text{1}}t}$. In this case, the resulting expression for $R_{\text{0}}$ is as follows:


$$\begin{array}{cc} R_{\text{0}} & =\;\frac{\beta }{\delta_{\text{s}}+\delta_{\text{c}}+\gamma }\left(\text{1}+\frac{\left(\text{1}-\varepsilon \right)}{\delta_{\text{s}}+\delta_{\text{c}}+\gamma }\left(\delta_{\text{s}}-\delta_{\text{c}}-\frac{{\text{2}\delta }_{\text{s}}\delta_{\text{c}}}{\delta_{\text{s}}+\delta_{\text{c}}+\gamma }\right)\right)\left(\text{1}-\frac{K\left(-\delta_{\text{s}}-\delta_{\text{c}}-\gamma \right)}{\mu }\right)\\ & +\beta (\text{1}-\varepsilon )\left(\left(\frac{\delta_{\text{s}}\delta_{\text{c}}}{\delta_{\text{s}}+\delta_{\text{c}}+\gamma }-\delta_{\text{s}}\right)\frac{K^{\prime }\left(-\delta_{\text{s}}-\delta_{\text{c}}-\gamma \right)}{\mu }+\left(\delta_{\text{c}}+\frac{\delta_{\text{s}}\delta_{\text{c}}}{\delta_{\text{s}}+\delta_{\text{c}}+\gamma }\right)\frac{\mu^{\text{2}}+\sigma^{\text{2}}}{\text{2}\mu }\right) \end{array}$$ (44)

We also use simplified expressions for other special cases of the modeled intervention. First, with surveillance and contact precautions as the only intervention component, i.e., no decolonization, we have $\gamma_{\text{d}}=\gamma$ and $R_{\text{0}}$ simplifies to:


$$R_{\text{0}}=\frac{\beta \varepsilon }{\delta_{\text{s}}+\delta_{\text{c}}+\gamma }\left(\text{1}-\frac{K\left(-\delta_{\text{s}}-\delta_{\text{c}}-\gamma \right)}{\mu }\right)+\frac{\beta (\text{1}-\varepsilon )}{\delta_{\text{c}}+\gamma }\left(\frac{\gamma }{\delta_{\text{c}}+\gamma }\left(\text{1}-\frac{K\left(-\delta_{\text{c}}-\gamma \right)}{\mu }\right)+\delta_{\text{c}}\frac{\mu^{\text{2}}+\sigma^{\text{2}}}{\text{2}\mu }\right)$$ (45)

Second, when decolonization is applied to surveillance-detected patients, and the clearance effect is assumed to be instantaneous ($\gamma_{\text{d}}\to \infty$), we can use the result from Model 2 with γ replaced by the sum of γ and $\delta_{\text{s}}$. That is,


$$R_{\text{0}}=\frac{\beta }{\delta_{\text{s}}+\delta_{\text{c}}+\gamma }\left(\left(\text{1}-\frac{\delta_{\text{c}}(\text{1}-\varepsilon )}{\delta_{\text{s}}+\delta_{\text{c}}+\gamma }\right)\left(\text{1}-\frac{K\left(-\delta_{\text{s}}-\delta_{\text{c}}-\gamma \right)}{\mu }\right)+\delta_{\text{c}}(\text{1}-\varepsilon )\frac{\mu^{\text{2}}+\sigma^{\text{2}}}{\text{2}\mu }\right)$$ (46)

### 3.3 Calibration of Model 3 to pre-intervention CPE in LTACH

To calibrate a model to length of stay data, we previously derived the following general formula for the cumulative distribution function, $F_{*}(t)$, for the time between admission and facility removal at equilibrium [5,23]:


$$F_{*}(t)=\text{1}-1^{\text{T}}e^{-𝐖t}{𝐱}_{\text{a}}\left(\text{1}-F(t)\right)$$ (47)

The function F(t) is the cumulative distribution function associated with the time-dependent discharge hazard h(t). In models where h(t)=0, and facility removal is modeled by state-dependent constant removal terms, the quantity $1^{\text{T}}e^{-𝐖t}{𝐱}_{\text{a}}$ is the fraction of patients who are still in the facility at time t after admission, with admission state distribution ${𝐱}_{\text{a}}$. Conversely, if there are no facility removal terms within W, $1^{\text{T}}e^{-𝐖t}{𝐱}_{\text{a}}=\text{0}$ and the above formula reduces to $F_{*}(t)=F(t)$. See our prior study for an example of a model with both nonzero h(t) (representing live discharge) and nonzero removal elements within W (representing death) [23].

For Model 3, we have $F_{*}(t)=F(t)$, and assume a mixed exponential-gamma distribution, with a portion $p_{x}$ of patients following an exponential distribution with rate $r_{x}$ and the rest following a gamma distribution with rate $r_{g}$ and shape parameter k. The following length of stay statistics reported in the LTACH study were used to estimate the 4 parameters: the mean, median, 25^th^ and 75^th^ percentile of the length of stay distribution. With 4 unknowns fit to those 4 data points (S1 Text), we calculated the unique solution (Table 3).

**Table 3:** Model calibration and $R_{\text{0}}$ results: CPE LTACH pre-intervention.

Parameter	Estimated value (95% CI)
Exponential length of stay probability ($p_{x}$)	0.580
Exponential length of stay rate parameter ($r_{x}$)	0.0285 per day
Gamma length of stay rate parameter ($r_{g}$)	0.179 per day
Gamma length of stay shape parameter (k)	5.74
Base transmission rate (β)	0.051 (0.043–0.059) per day
Clinical detection rate ($\delta_{\text{c}}$)	0.0084 (0.0073 – 0.0097) per day
Facility basic reproduction number ($R_{\text{0}}$)	1.24 (1.04–1.45)

For the CPE parameters, under our assumed values for three independently based parameters (ε=0.5, γ=1/ 387 per day, σ=0.85), we estimated the pre-intervention values of β, $\delta_{\text{c}}$, and $R_{\text{0}}$ from CPE data in LTACHs, using Model 3 (Table 3). Our estimate for the baseline transmission rate, β, was 0.051 per day (95% range 0.043 to 0.059). The estimate of rate of progression to clinical detection, $\delta_{\text{c}}$, was 0.0084 per day (0.0073 to 0.0097). Using those results and our formula for Model 3 above, we estimated $R_{\text{0}}$ = 1.24 (1.04 to 1.45).

### 3.4 Application of Model 4 to vertical intervention analysis for CPE in LTACH

**Fig 1:**
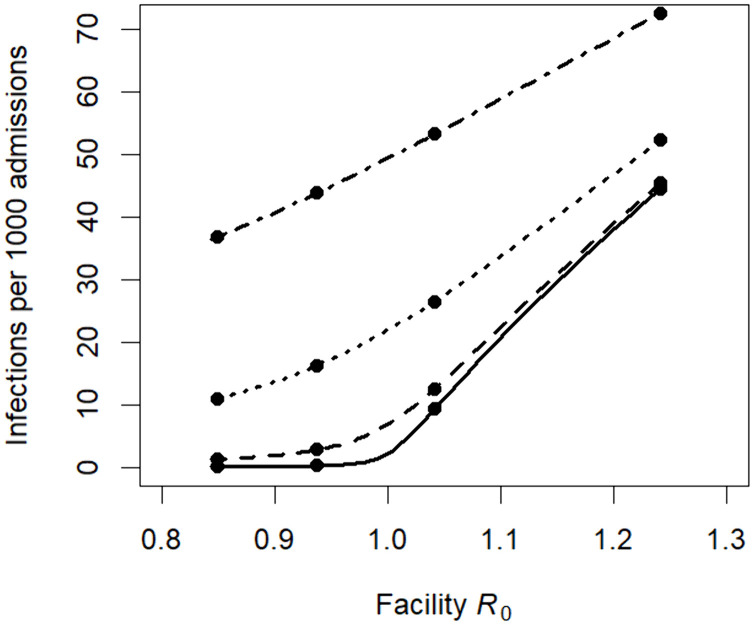
Relationships between facility ${𝐑}_{\text{0}}$ and equilibrium clinical infection incidence with constant importation. The curve was generated using formulas for Model 4, at values for the surveillance test frequency ranging from 0 to once per week and no pathogen reduction effect of the intervention. Other model parameters were set at the assumed and estimated values described in Section 3.3, fit to data from CPE in LTACHs. The points (circles) represent average surveillance intervals of, from left to right, one week, two weeks, one month, and no surveillance. Facility ${𝐑}_{\text{0}}$ was calculated using the formula in Section 3.2.4 with no decolonization, and we calculated infections per admission as the product of the mean length of stay, the per capita clinical detection rate, and the equilibrium prevalence of colonized, non-clinically detected patients, assuming a constant importation rates of 0.01% (solid), 0.1% (dashed), 1% (dotted), and 5% (dash-dot).

First, we estimated the effect of the biweekly CPE surveillance component of the intervention reported in the source study. Using the formula for $R_{\text{0}}$ from Model 4, with $\gamma_{\text{d}}=\gamma$ (no decolonization effect) and surveillance detection rate $\delta_{\text{s}}=\text{0}.\text{058}$ per day (once per 14 days multiplied by scaling factors for imperfect compliance and imperfect test sensitivity). With these values, and with other assumptions unchanged from the pre-intervention scenario modeled in the previous section, we estimated $R_{\text{0}}$ = 0.94 (95% range 0.79 to 1.08). Increasing the frequency of surveillance from biweekly to weekly decreases the estimate to $R_{\text{0}}$ = 0.85 (0.72 to 0.98). The minimum frequency of surveillance testing required to make $R_{\text{0}}<\;\text{1}$ was once per 3.2 weeks (1.1 to 23 weeks). We plotted the relationship between $R_{\text{0}}$ at different frequencies of surveillance and the number of clinical infections per 1000 admissions at equilibrium, assuming the admission rate of CPE carriers remains constant at 0.01%, 0.1%, 1%, and 5% (Fig 1). When there is continual importation from external sources, reducing $R_{\text{0}}<\;\text{1}$ does not eliminate infections and does not exhibit a noticeable threshold effect when the importation rate is higher. However, when the importation rate is low, the plotted relationship demonstrates an inflection point at the $R_{\text{0}}=\text{1}$ threshold, where the infection incidence rises much more steeply with increasing $R_{\text{0}}$ when it is above the threshold.

Next, we modeled the effect of combining surveillance with decolonization via pathogen reduction treatment of surveillance-detected patients, represented by increasing the non-treated rate of clearing colonization among of surveillance-detected patients by a factor f> 1, such that $\gamma_{\text{d}}=f\gamma$. For different values of f, we calculated $R_{\text{0}}$ for weekly and biweekly surveillance, and the surveillance rate required to achieve $R_{\text{0}}<\;\text{1}$ (Table 4). For example, with f=5 (five-fold increase in clearance rate due to the pathogen reduction treatment), the $R_{\text{0}}$ estimate with biweekly surveillance was 0.87 (0.73 to 1.00), an 8% decrease from the estimate of 0.94 with no decolonization effect. The upper bound of the decolonization factor (f→∞, or immediate clearance upon detection) achieves $R_{\text{0}}=\text{0}.\text{56}$ (0.47 to 0.64) with biweekly surveillance. With immediate clearance, a surveillance interval of once per 10 weeks (6–56 weeks) could potentially achieve $R_{\text{0}}<\;\text{1}$.

**Table 4:** $R_{0}$ and threshold estimates for combined surveillance and decolonization intervention.

Decolonization rate factor	Mean days to decolonization	$R_{0}$ with weekly surveillance (95% CI)	$R_{0}$ with biweekly surveillance (95% CI)	Surveillance interval needed to achieve $R_{0}$ < 1 (95% CI)
1 (no effect)	387	0.85 (0.72, 0.98)	0.94 (0.79, 1.08)	3.2 weeks (1.1, 23)
2	194	0.82 (0.70, 0.95)	0.92 (0.78, 1.06)	3.6 weeks (1.4, 25)
5	77	0.76 (0.64, 0.87)	0.87 (0.73, 1.00)	4.5 weeks (2.0, 30)
10	39	0.68 (0.58, 0.79)	0.81 (0.69, 0.93)	5.6 weeks (2.7, 35)
50	8	0.49 (0.42, 0.56)	0.66 (0.56, 0.76)	8.4 weeks (4.5, 48)
100	4	0.43 (0.37, 0.50)	0.62 (0.52, 0.71)	9.2 weeks (5.0, 51)
∞ (immediate)	0	0.36 (0.31, 0.41)	0.56 (0.47, 0.64)	10.3 weeks (5.7, 56)

### 3.5 Length of stay intervention analysis

The parameters of Model 3 governing removal (death or discharge) rates of patients were calibrated to LTACH length of stay statistics (Table 1), giving rise to an overall length of stay distribution with mean μ=33.8 days and standard deviation σ=28.1 days. With the mixture model we used to govern removal, a fraction $p_{x}=\text{0}.\text{580}$ of admitted patients follow an exponentially distributed length of stay (constant discharge-or-death hazard), with mean and standard deviation $\mu_{x}=\sigma_{x}=\;\text{35}.\text{1}$ days. The remaining patients follow gamma-distributed length of stay with shape parameter k>1 (meaning the removal hazard increases with time of stay), which gives a length of stay distribution with mean $\mu_{g}=\text{32}.\text{0}$ days and standard deviation $\sigma_{g}=\text{13}.\text{4}$ days. Thus, the patients following the exponential distribution have a slightly higher mean and much higher variance in length of stay than the other group. We refer to the patients following the exponentially distributed length of stay as the high-variance patients and the patients following the gamma-distributed length of stay as the low-variance patients.

**Fig 2:**
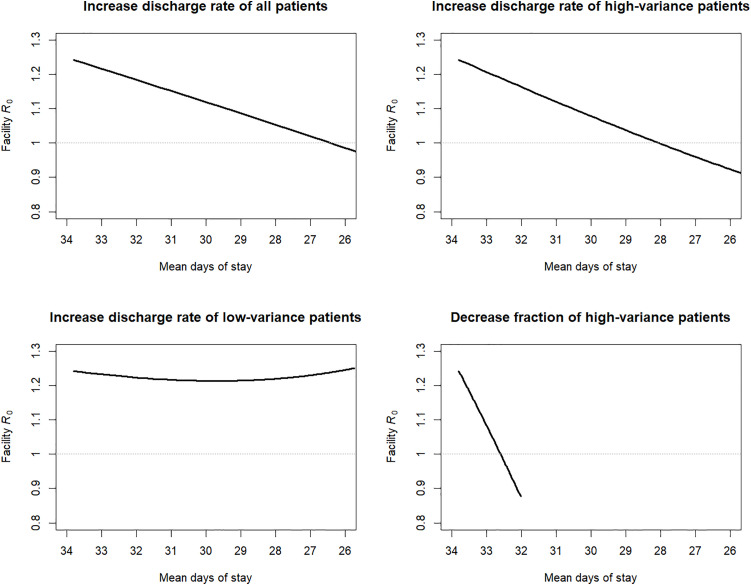
Effect of length-of-stay interventions on facility basic reproduction number. Calculations of the facility reproduction number, ${𝐑}_{\text{0}}$, for Model 3 with the following fixed values calibrated to pre-intervention CRE data in long-term acute care hospitals (Table 3): transmission rate β= 0.051 per day, clinical detection rate $\delta_{\text{c}}=\;\text{0}.\text{0084}$ per day, colonization clearance rate γ=1/ 387 per day, and contact precaution effectiveness ϵ=0.5. Length of stay was governed by a 4-parameter mixed exponential–gamma distribution, and specific parameters were varied from the baseline values (Table 3) in a direction that decreased the overall mean length of stay from left-to-right in each of the sub-figures, while holding all other parameters constant. Upper-left: both the exponential rate parameter ${𝐫}_{\text{x}}$ and the gamma rate parameter ${𝐫}_{\text{g}}$ were simultaneously increased by the same factor from their baseline values. Upper-right: the exponential rate parameter ${𝐫}_{\text{x}}$ was increased from its baseline value. Lower-left: the gamma rate parameter ${𝐫}_{\text{g}}$ was increased from its baseline value. Lower-right: the fraction of patients following the exponential distribution ${𝐩}_{\text{x}}$ was decreased from its baseline value.

We tested the effects of interventions that alter different length of stay parameter values on $R_{\text{0}}$ for Model 3 calibrated to pre-intervention LTACH CPE data (Fig 2). We chose four interventions that decrease the mean length of stay from the baseline pre-intervention 33.8 days. First, when increasing the discharge rate of all patients (i.e., increasing the rate parameters $r_{x}$ and $r_{g}$ by the same factor), $R_{\text{0}}$ decreases and crosses the $R_{\text{0}}<\text{1}$ threshold as the mean length of stay drops below about 26 days. Second, when increasing the discharge rate $r_{x}$ of the high-variance patients only, $R_{\text{0}}$ decreases below 1 as the mean length of stay drops to about 28 days. Third, when increasing the discharge rate $r_{g}$ of the low-variance patients, $R_{\text{0}}$ does not decrease below 1.2 and in fact begins increasing as the mean length of stay drops below about 29 days and lower. Fourth, when decreasing the fraction $p_{x}$ of patients following the high-variance exponential distribution, $R_{\text{0}}$ drops rapidly and falls below 1 as the mean length of stay drops below about 32.6 days. When $p_{x}=\text{0}$ (all patients follow the low-variance gamma distribution with mean 32.0 days), $R_{\text{0}}=$ 0.88.

**Fig 3:**
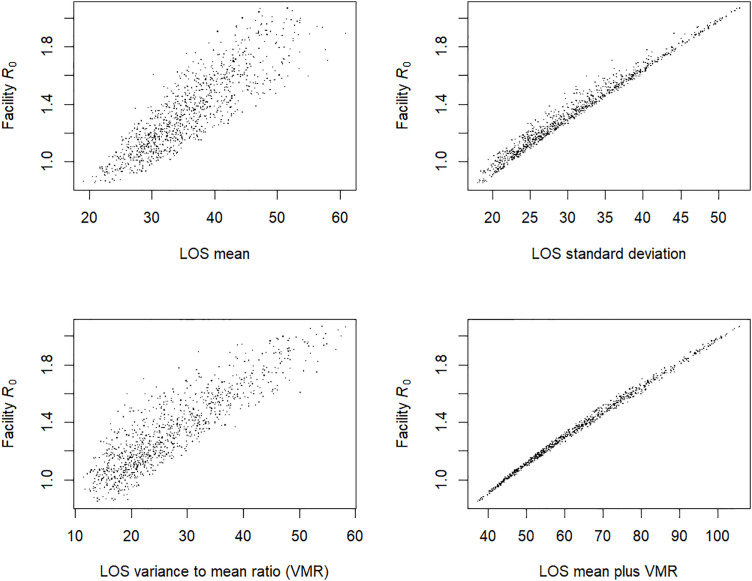
Scatterplot of facility basic reproduction number vs. length of stay (LOS) distribution statistics. Calculations of the facility reproduction number, ${𝐑}_{\text{0}}$, for Model 3 with the following fixed values calibrated to pre-intervention CRE data in long-term acute care hospitals (Table 3): transmission rate β=0.051 per day, clinical detection rate $\delta_{\text{c}}=\text{0}.\text{0084}$ per day, colonization clearance rate γ=1/ 387 per day, and contact precaution effectiveness ϵ=0.5. The dots represent ${𝐑}_{\text{0}}$ results for 1000 sets of values for each of the four parameters governing the mixed exponential–gamma length of stay distribution, drawn from four independent uniform distributions ranging from 60% to 140% of the calibrated values in Table 3. Each subplot displays the same 1000 ${𝐑}_{\text{0}}$ results, plotted against different statistics of the length of stay distribution for each set of length-of-stay parameters.

As the results in Fig 2 demonstrate that the mean length of stay did not necessarily correlate with $R_{\text{0}}$, we tested the relationship between $R_{\text{0}}$ and other statistics for the length of stay distribution (Fig 3). The dots in the scatterplot represent the results from randomly chosen combinations of the four length-of-stay parameters $p_{x}$, $r_{x}$, $r_{g}$, and k, each picked from independent uniform distributions ranging from plus-or-minus 40% of the values from the model calibrated to the LTACH data (Table 3). For each of the random set of parameter values, we calculated $R_{\text{0}}$ and the mean, standard deviation, variance-to-mean ratio (VMR) and the sum of the mean and VMR. The latter statistic was chosen because of its appearance in our result for the $R_{\text{0}}$ formula for Model 1.

The length of stay mean produced a weaker correlation with $R_{\text{0}}$ (Pearson’s correlation coefficient PCC = 0.857) compared to the correlation between the standard deviation and $R_{\text{0}}$ (PCC = 0.986). The VMR produced PCC = 0.860, and the sum of the mean and VMR produced the strongest correlation with $R_{\text{0}}$, with PCC = 0.997 and the relationship appearing nearly linear (Fig 3).

Files with code that produces results in Figs 1–3 and Tables 3 and 4 are available on the facilityepimath GitHub repository at https://github.com/EpiForeSITE/facilityepimath/tree/v0.2.0/inst.

## 4 Discussion

This study provides a flexible, quantitative framework for calculating the basic reproduction number $R_{\text{0}}$ for a wide range of mathematical models of transmissions among patients admitted to healthcare facilities. We showed that such calculations can provide actionable insights for understanding the factors leading to certain facilities reaching $R_{\text{0}}>\text{1}$ for high-priority pathogens, creating a setting where a single patient importation can lead to a sustained outbreak. We demonstrated that one set of high-risk facilities plausibly produced $R_{\text{0}}>\text{1}$ for CPE, a multi-drug-resistant organism of high concern, and analyzed models that provided nuanced insights into alternate interventions that could reduce $R_{\text{0}}$ in such facilities to below threshold.

A facility with $R_{\text{0}}>\text{1}$ is at risk not only of explosive outbreaks among its own patients, but also for acting as a transmission amplifier within a regional healthcare network [30]. Patients needing care in a high-risk (and high $R_{\text{0}}$) facility like an LTACH may be likely to subsequently require care in other hospitals or long-term care facilities, and thus they pose risk of continued spread of pathogens they had acquired in the LTACH. Prior simulation studies of CPE transmission among patients in a regional network of healthcare facilities, including short-stay hospitals, nursing homes and LTACHs, demonstrated that interventions including efforts focused on detecting carriage among LTACH patients could dramatically reduce CPE infections over the whole region [4].

Those prior findings of a dramatic decrease in simulated CPE infections due to LTACH-based intervention are consistent with a theoretical result we produced here: realistic rates of surveillance for CPE carriage and contact precautions for detected CPE carriers in an LTACH can plausibly move $R_{\text{0}}$ from above to below the critical threshold of 1. Crossing that threshold can explain the disproportionately high benefit achieved by a moderate increase in mitigation effort observed in simulation studies. A patient population health benefit that increases non-linearly with increasing intervention cost can also manifest in a highly efficient, cost-saving resource allocation, as predicted by a health economic analysis of a model of a CPE decolonization intervention in LTACHs [5]. However, our results in Fig 1 demonstrate one caveat to this insight: a facility with continually high rates of importation may not experience the same threshold-crossing benefit as a facility where colonized patients were more largely self-generated. For example, in a healthcare system with multiple $R_{\text{0}}>\text{1}$ facilities linked by patient exchange, a single healthcare facility reducing $R_{\text{0}}<\text{1}$ would not experience the full benefits of threshold effect if it still exists in an above-threshold system.

Our result for the effect of CPE surveillance on reducing facility $R_{\text{0}}$ was determined by the effectiveness of contact precautions to reduce transmission from detected carriers and rate of mid-stay surveillance only. The rate of *admission* surveillance does not appear in the $R_{\text{0}}$ formula, because we defined $R_{\text{0}}$ as the expected number of transmissions from a patient who acquires CPE after admission, between the time of acquisition and discharge or death. While admission surveillance can reduce the chances that otherwise undetected CPE importers will set off a chain of transmissions, nothing short of perfect admission detection and 100% effective contact precautions could entirely prevent a within-facility transmission from occurring. If any transmissions occur in a facility with $R_{\text{0}}>\text{1}$ by our definition, admission surveillance would not affect continuing circulation of CPE among in-patients who were each non-carriers at admission.

This insight exhibits why the admission reproduction number (defined $R_{A}$ in many studies), the expected number of transmissions from an admitted colonized patient, is not necessarily equal to $R_{\text{0}}$, the expected number of transmissions from a colonized patient who acquired colonization after admission. If $R_{A}\neq R_{\text{0}}$, then $R_{A}>\text{1}$ is not the correct threshold condition for characterizing outbreak risk. The implications of this finding are potentially important for facilities considering implementing a surveillance intervention: testing patients during the middle of their stay is the critical component that reduces the chances of a facility being a transmission amplifier. A caveat to this insight is that a broader definition of $R_{\text{0}}$ would cover a patient’s entire period of colonization, which might include multiple different stays at healthcare facilities. Admission surveillance, particularly for patients having recently stayed at the same or another high-risk facility, would help reduce $R_{\text{0}}$ under that extended model.

For the single-stay facility model, we showed that the biweekly rate of surveillance for CPE reported in a Chicago area LTACH intervention [21] may have been sufficient to reduce $R_{\text{0}}$ from above to below threshold, assuming 50% effectiveness of contact precautions at reducing transmission from detected patients, 95% adherence to surveillance testing, and 85% sensitivity of the test. Our $R_{\text{0}}$ formula provides a quick method for testing sensitivity of $R_{\text{0}}$ estimates to those assumptions as well as the rate of surveillance as an intervention decision variable.

In addition to contact precautions for identified colonized patients, the pathogen reduction intervention we tested represents the administration of treatment to detected carriers that would decolonize the patient or act to increase the rate of clearance by some mechanism, a class of treatments that has recently garnered attention [20]. Our prior modeling study demonstrated the potential for high cost-effectiveness that a drug with rapid decolonization effects could have when administered in a high-transmission facility to even a small portion of colonized patients [5]. Our results here bolster the theoretical underpinnings of that prior result and can help identify scenarios and settings where new drugs could help achieve transmission threshold effects that produces a disproportionately high health benefit. Our model combining surveillance and decolonization demonstrates a synergistic effect of the two strategies when the drug can achieve clearance rapidly.

The feasibility of implementing such a combined surveillance-and-decolonization intervention would depend on the method of pathogen reduction being used to achieve decolonization. For organisms like CPE and other pathogenic bacteria that colonize the gut, treatments with potential pathogen reduction agents are in early stages of conception, development, testing, or approval, such as selective decontamination of the digestive tract [31], bacteriophages [32], or probiotic treatments that could fortify the human microbiome’s natural ability to clear harmful colonization [33]. As the mechanisms, costs, and timings of such treatments become clearer, our model could be refined to produce cost-effectiveness analyses tailored to a particular approach.

As with any epidemiological modeling study attempting to project intervention effects in a real-world setting, our results depend on assumptions that may not be accurate. While we matched our assumptions to published data wherever possible, some key parameter value assumptions did not have strong data-based justification. For example, the relative transmissibility of detected CPE carriers compared to undetected carriers in hospitals is unknown, as the transmitter to an acquisition is rarely identified definitively during CPE outbreaks. In addition, given that the decolonization drug explored in our model is hypothetical, modeling its effects relative to the also-uncertain natural clearance rate is largely based on guesswork. Nevertheless, our equation-based results allow for quick exploration of alternate assumptions, and such sensitivity analyses in this and prior work [5,23] suggest that our findings for these intervention effects are largely robust.

Another major finding of this study is a characteristic property of the length of stay distribution of facility patients that largely governs its relationship to $R_{\text{0}}$. Namely, $R_{\text{0}}$ was directly proportional to the ratio of the *second raw moment* of the length of stay distribution to the mean of the distribution. The second raw moment is the average of the squares of each individual length of stay and is also equal to the sum of the variance and the square of the mean. Thus, the characteristic ratio can also be expressed as the sum of the variance and the variance-to-mean ratio of the distribution. This is an exact result for Model 1, where colonized patients remain colonized from the time of acquisition to discharge or death with no change in their transmissibility. For more complicated models, the proportionality is not exact, as the $R_{\text{0}}$ formulas depend on higher moments of the length of stay distribution via the moment-generating function. But we showed in Fig 3 that the characteristic ratio was nevertheless an excellent predictor of changes to $R_{\text{0}}$ due to changes in a complicated length of stay distribution model.

Intuition as to why the square of lengths of stay is a characteristic property governing facility $R_{\text{0}}$ lies in understanding the length of stay of a typical patient who might acquire colonization while they are an in-patient at a healthcare facility. If a random patient residing in the facility on a given day becomes colonized, their time remaining in the facility before discharge or death is the characteristic time duration governing their subsequent potential to transmit to others. Calculating the average length of stay of a cross-section of patients in the facility at a given time is different than calculating the average length of stay of a set of admitted patients, because longer-stay patients are overrepresented in the cross-section due to their taking up facility bed spaces for longer. Specifically, in a sample of patients in the facility at a given time, patients with a longer length of stay are more likely to be sampled, in direct proportion to their length of stay. For example, a patient with a length of stay twice as long as another was twice as likely to be in the facility on the day of sampling. Therefore, the mean length of stay of the patients sampled is a weighted mean of the actual lengths of stays of all admitted patients, where the weights are the length of stay. The weighted mean of lengths of stay weighted by length of stay reproduces the characteristic quantity: the mean of the square of length of stay divided by the mean.

This finding is an example of well-known result in theoretical ecology of infectious disease [34]. In applications, the sum of the mean and the variance-to-mean ratio of a transmission-relevant quantity being proportional to $R_{\text{0}}$ has been shown in a variety contexts, including the number of sexual partners per person infected with sexually transmitted disease [35], the biting rates of mosquitos transmitting disease to humans [36], and household sizes for transmission through a community via housemates [37]. Our finding that this result also applies to facility length of stay for healthcare-associated infections is, to our knowledge, a novel contribution to the field of healthcare transmission modeling.

The practical implications of this result are that both the mean and the variance of the length of stay distribution are important for characterizing facility outbreak risk, and both should be more regularly calculated and considered by facilities undergoing transmission studies. Reported facility length of stay statistics often include only the mean and/or quantiles such as the median or inter-quartile range, which fail to uniquely identify the variance. Individual facilities and facility types vary widely in their length of stay patterns. Aside from LTACHs, other types of long-term care facilities such as nursing homes or skilled nursing facilities may produce different quite different average and variation in length of stay compared to our example, and those patterns may rapidly and unevenly fluctuate due to shifting policies and other factors [38]. Short-stay hospitals generally have a much lower mean length of stay that reduces the risk of sustained transmission after a single importation, but some hospitals could have an unusually high length of stay variance due to a subset of long-stay patients, which would put them at greater risk than the mean length of stay alone would indicate. Our findings produce a unifying framework for analyzing and comparing facilities with widely different and complicated length of stay patterns: the sum of the mean and the variance-to-mean ratio in length of stay correlates with $R_{\text{0}}$ and thus with outbreak risk.

Furthermore, we showed that the effect of efforts to reduce lengths of stay as a transmission risk-reduction intervention are not always well characterized by the intervention’s effect on the mean. If the mean length of stay is reduced in a way that simultaneously increases the variance-to-mean ratio, it is possible that facility outbreak risk could increase, as measured by $R_{\text{0}}$. For LTACHs and similar facilities that specialize in care for high acuity patients, it might be possible to characterize the condition and care needs of incoming patients to estimate not only their expected length of stay, but also the uncertainty (i.e., variance) in days of care they will require. If a facility can control the composition of admitted patients and/or target length of stay reduction efforts on certain groups, efforts to reduce the fraction of the high-variance group of patients or to increase their discharge rates would be favored for reducing facility outbreak risk.

Our general model has capabilities for investigating additional nuances of length of stay effects, including use of state-dependent discharge or death rates via the $\omega_{i}$ removal terms. To reduce unnecessary complexity in our examples, we set $\omega_{i}=\text{0}$ and focused on using the state-independent removal hazard *h*(*t*) to model and calibrate the length of stay distribution. Our assump*t*ion ignores potential correlation between patient states and discharge and death hazard. For example, patients experiencing symptomatic infection could have a reduced or eliminated discharge hazard and a higher death rate, and patients with more serious underlying conditions may have both a lower discharge hazard and a higher per-exposure susceptibility than other patients. Calibrating a model with these details to length of stay statistics would require more care but is possible under our general framework, as shown in our prior work [5,23].

We have released an R package, facilityepimath [29], that includes a function to calculate $R_{\text{0}}$ numerically, using the general equation we derived, for a wide variety of models. The package contains example code reproducing the results for the models we investigated in this manuscript, as well as example demonstrations of how other users can use the function to calculate $R_{\text{0}}$ for any model that fits our broad general framework, including models with nuanced assumptions governing facility length of stay. The package also provides other functions, e.g., to calculate the equilibrium of a model for a given, constant rate of importation of infectious individuals to the facility, which is useful for calibrating models to observed levels of admission and cross-sectional prevalence, as done in this work, and for assessing the impacts of transmission-reducing interventions under ongoing importation [5,23].

While our formula applies to a wide range of possible models, there are limitations to what can be assumed. For contact assumptions affecting transmission, the model form requires that all susceptible compartments have the same relative exposure to individuals in different colonized compartments. Therefore, our results do not apply to segregated contact models in which, for example, the compartments represent patients in different wards of a healthcare facility with different within-ward vs. cross-ward contact rates. Our model also does not include the effects of transmission during an episode of infectiousness extending beyond a single facility stay. Extensions to multiple facility stays via readmission to the same facility and transmission in multiple different facilities or in the community outside a healthcare facility are potential subjects of future work. Our model parameter estimates from CPE data were possible because the facilities observed steady state levels of importation and cross-sectional prevalence, which made our equilibrium assumption reasonable. Fitting parameters to facilities observing more transient patient state dynamics would require additional model components and solution techniques, as would studying the transient dynamics toward a new equilibrium after introducing an intervention. With a deterministic framework, our model also does not quantify stochastic variation in outbreaks stemming from a single introduction. In a stochastic model simulation, a sustained chain of transmission after a single introduction would have a much higher probability of occurring when $R_{\text{0}}>\text{1}$, but would not be guaranteed. With high variability in factors affecting transmission, a facility with $R_{\text{0}}>\text{1}$ might experience many introductions leading to only small outbreaks before a large outbreak eventually takes hold [39].

In conclusion, we have derived a novel, general mathematical result for theoretical quantities that have important implications for study and control of high-priority infectious disease health threats. Our findings for healthcare facility transmission models could be applicable to other at-risk settings with similarly rapid population turnover. Locations with above-threshold transmission conditions can pose a significant risk to population health, while at the same time presenting opportunities for targeted interventions that are highly efficient. Seeking a nuanced understanding of those conditions and their relationship to intervention mechanisms should be a component of strategic planning for public health efforts.

## Supporting information

S1 Text Supplementary methods and results. (PDF)
