# Supplementary material for: Transmission thresholds for the spread of infections in healthcare facilities
Source: PLoS Comput Biol. 2025 Oct 15;21(10):e1013577. doi: 10.1371/journal.pcbi.1013577 (PMC12539749; doi:10.1371/journal.pcbi.1013577)
Supplement: S1 Text — (PDF) [file pcbi.1013577.s001.pdf]

Supporting Information for "Transmission thresholds for the spread of infections in healthcare facilities"

S1. Supplementary Methods and Results

Length of stay model calibration to LTACH data

We assume that patient length of stay is a random variable with cumulative distribution function $F(t)$. The source LTACH study (21) provides the mean and 25th, 50th, and 75th percentiles of the overall length of stay distribution (μ, l_{25}, l_{50}, l_{75}).

For fitting Model 3 to data, we have four equations constraining the length of stay distribution:

$$\mu = \int_0^\infty \big(1 - F(\tau)\big)d\tau, \qquad 0.25 = F(l_{25}), \qquad 0.5 = F(l_{50}), \qquad 0.75 = F(l_{75})$$

We assume a mixed exponential-gamma distribution for the length of stay distribution, with a portion p_x of patients following an exponential distribution with rate r_x and the rest following a gamma distribution with rate r_g and shape parameter k. For this distribution:

$$F(t) = p_x(1 - e^{-r_x t}) + (1 - p_x)\frac{1}{\Gamma(k)}\int_0^{r_g k t} \tau^{k-1} e^{-\tau} d\tau$$

$$\mu = p_x/r_x + (1 - p_x)k/r_g$$

We apply these two functions to the four equations above and numerically solve for the four unknown parameters p_x, μ_x, μ_g, and k.

From the LTACH study (21), we have $\mu = 33.8$ days, $l_{25} = 16$ days, $l_{50} = 28$ days, and $l_{75} = 43$ days, which leads to:

$$p_x = 0.580,\ r_x = 0.0285 \text{ per day},\ r_g = 0.179 \text{ per day, and } k = 5.74$$

Equilibrium calibration to LTACH CPE data

As described in the main text, we calibrated the patient acquisition rate α at equilibrium and the per-capita clinical detection rate δ_c of a colonized patient to data describing the cross-sectional prevalence of CPE carriage and the overall CPE clinical detection incidence rate observed at the LTACHs (21). We assumed that Model 3 (main text) was at equilibrium with the observed constant CPE admission positivity rate. Therefore, we required a formula for the equilibrium prevalence of the three patients states of Model 3 at the given initial conditions (admission states). Here we derive a formula for the equilibrium of a general model, then apply the formula to Model 3.

Let $\mathbf{x}(t)$ be the vector of probabilities that a patient is alive, not discharged, and in each state x_i at time t after admission. We have the system

$$\frac{d\mathbf{x}}{dt} = \mathbf{W}\mathbf{x} - h(t)\mathbf{x}, \qquad \mathbf{x}(0) = \mathbf{x}_\mathrm{a}.$$

Here, $\mathbf{W}$ contains state-to-state transition rates and state-specific facility removal rates during the stay. The hazard function $h(t)$ is the removal rate at time t of the stay, where the same function applies to every state. The vector $\mathbf{x}_\mathrm{a}$ is the distribution of states at admission. The solution to this equation is expressed

$$\mathbf{x}(t) = e^{\int_0^t h(\tau)d\tau} e^{\mathbf{W}t}\mathbf{x}_\mathrm{a}.$$

If we express the hazard function as that of a probability distribution: $h(t) = f(t)/\left(1 - F(t)\right)$, where f and F are the probability density function and cumulative distribution function of the same distribution, then we can express $\mathbf{x}(t)$ as

$$\mathbf{x}(t) = \left(1 - F(t)\right)e^{\mathbf{W}t}\mathbf{x}_{\mathrm{a}}.$$

If there are no discharge or death rates within the $\mathbf{W}$ matrix, then F is the cumulative distribution function of the facility length of stay distribution.

If we assume that patients are continually admitted to the facility at a constant rate of q patients per time, *ad infinitum*, then the expected number of patients in the facility between stay-time t_1 and t_2 in each state is $\int_{t_1}^{t_2} \mathbf{x}(t) q dt$. So, the expected total number of inpatients in each state is given by the vector

$$\mathbf{n}^* = \int_0^\infty q\left(1 - F(t)\right)e^{\mathbf{W}t}\mathbf{x}_{\mathrm{a}} dt$$

And the expected total number of patients in the facility is $\mathbf{N}^* = \mathbf{1}^{\mathrm{T}}\mathbf{n}^*$. Then, the equilibrium cross-sectional state distribution within the facility is $\mathbf{x}^* = \mathbf{n}^*/\mathbf{N}^*$, or

$$\mathbf{x}^* = \frac{\int_0^\infty \left(1 - F(t)\right)e^{\mathbf{W}t}\mathbf{x}_{\mathrm{a}} dt}{\mathbf{1}^{\mathrm{T}} \int_0^\infty \left(1 - F(t)\right)e^{\mathbf{W}t}\mathbf{x}_{\mathrm{a}} dt}$$

To evaluate the integral in this expression, we must consider the integral $\int_0^\infty \left(1 - F(t)\right)\varphi_i(t) dt$ for the eigenfunctions φ that comprise the elements of the matrix exponential $e^{\mathbf{W}t}$. If the state dependent removal rates ω_i are all nonzero, the eigenvalues λ_i of $\mathbf{W}$ are negative and real. The eigenfunctions associated with non-degenerate eigenvalues then take the form $\varphi_i(t) = e^{\lambda_i t}$. We use integration by parts:

$$\int_0^\infty \left(1 - F(t)\right)e^{\lambda_i t} dt = \frac{1}{\lambda_i}\left[\left(1 - F(t)\right)e^{\lambda_i t}\right]_{t=0}^{t\to\infty} + \frac{1}{\lambda_i}\int_0^\infty e^{\lambda_i t} f(t) dt = \frac{1}{\lambda_i}(0 - 1) + \frac{1}{\lambda_i}M(\lambda_i) = \frac{M(\lambda_i) - 1}{\lambda_i}$$

Here, f and M are the probability density function and moment-generating function of the distribution associated with the removal hazard, respectively. For ease of notation, we define the function

$$K(x) = \frac{M(x) - 1}{x}$$

If there are any repeated, negative eigenvalues that are degenerate, there would be eigenfunctions of the form $\varphi_i(t) = t^r e^{\lambda_i t}$ for integer $r \geq 1$. We note that if $K(\lambda_i) = \int_0^\infty \left(1 - F(t)\right)e^{\lambda_i t} dt$ then $K^{(r)}(\lambda_i) = \int_0^\infty \left(1 - F(t)\right)t^r e^{\lambda_i t} dt$, where $K^{(r)}$ is the rth derivative of K, defined above.

Finally, for models where any state-dependent removal rates ω_i are set to zero, there may be an eigenvalue of $\mathbf{W}$ equal to zero, in which case there will be at least one eigenfunction $\varphi_i(t)$ that is constant. Then there will be a component of the $\int_0^\infty \left(1 - F(t)\right)\varphi_i(t) dt$ expression equal to the mean μ of the distribution for which $F(t)$ is the cumulative distribution function, as $\int_0^\infty \left(1 - F(t)\right) dt = \mu$.

In summary, the vector $\int_0^\infty \left(1 - F(t)\right)e^{\mathbf{W}t}\mathbf{x}_{\mathrm{a}} dt$ is constructed by calculating $e^{\mathbf{W}t}\mathbf{x}_{\mathrm{a}}$ and replacing the $t^r e^{\lambda_i t}$ terms in each vector element with $K^{(r)}(\lambda_i)$ and the constant terms with μ.

For Model 3, we have the following components:

$$\mathbf{x} = \begin{pmatrix} S \\ C \\ C_{\mathrm{cd}} \end{pmatrix}, \quad \mathbf{x}_{\mathrm{a}} = \begin{pmatrix} 1 - p_{\mathrm{a}} \\ p_{\mathrm{a}} \\ 0 \end{pmatrix}.$$

$$\mathbf{W} = \begin{pmatrix} -\alpha & \gamma & 0 \\ \alpha & -\gamma - \delta_{\mathrm{c}} & 0 \\ 0 & \delta_{\mathrm{c}} & 0 \end{pmatrix}$$

$$\mathbf{x}^* = \frac{\int_0^\infty \big(1 - F(t)\big) e^{\mathbf{W}t} \mathbf{x}_{\mathrm{a}} dt}{\mathbf{1}^{\mathrm{T}} \int_0^\infty \big(1 - F(t)\big) e^{\mathbf{W}t} \mathbf{x}_{\mathrm{a}} dt}$$

To calculate the matrix exponential $e^{\mathbf{W}t}$ we require the eigenvalues of $\mathbf{W}$. The first two eigenvalues are the eigenvalues of the upper-left 2-by-2 submatrix, and the third eigenvalue is 0. The first two eigenvalues λ_1 and λ_2 are:

$$\lambda_1 = \frac{-(\alpha + \gamma + \delta_{\mathrm{c}}) - \sqrt{(\alpha + \gamma + \delta_{\mathrm{c}})^2 - 4\alpha\delta_{\mathrm{c}}}}{2}$$

$$\lambda_2 = \frac{-(\alpha + \gamma + \delta_{\mathrm{c}}) + \sqrt{(\alpha + \gamma + \delta_{\mathrm{c}})^2 - 4\alpha\delta_{\mathrm{c}}}}{2}$$

Then, the matrix exponential is:

$$e^{\mathbf{W}t} = \begin{pmatrix} \frac{\alpha+\lambda_1}{\lambda_1-\lambda_2} e^{\lambda_2 t} - \frac{\alpha+\lambda_2}{\lambda_1-\lambda_2} e^{\lambda_1 t} & \frac{\gamma}{\lambda_1-\lambda_2}\left(e^{\lambda_1 t} - e^{\lambda_2 t}\right) & 0 \\ \frac{(\alpha+\lambda_1)(\alpha+\lambda_2)}{\gamma(\lambda_1-\lambda_2)}\left(e^{\lambda_2 t} - e^{\lambda_1 t}\right) & \frac{\alpha+\lambda_1}{\lambda_1-\lambda_2} e^{\lambda_1 t} - \frac{\alpha+\lambda_2}{\lambda_1-\lambda_2} e^{\lambda_2 t} & 0 \\ \frac{\delta_{\mathrm{c}}(\alpha+\lambda_1)(\alpha+\lambda_2)}{\gamma\lambda_1\lambda_2}\left(\frac{\lambda_1}{\lambda_1-\lambda_2} e^{\lambda_2 t} - \frac{\lambda_2}{\lambda_1-\lambda_2} e^{\lambda_1 t} - 1\right) & \frac{\delta_{\mathrm{c}}}{\lambda_1\lambda_2}\left(\frac{\lambda_2(\alpha+\lambda_1)}{\lambda_1-\lambda_2} e^{\lambda_1 t} - \frac{\lambda_1(\alpha+\lambda_2)}{\lambda_1-\lambda_2} e^{\lambda_2 t} + \alpha\right) & 1 \end{pmatrix}$$

$$e^{\mathbf{W}t}\mathbf{x}_{\mathrm{a}} = \begin{pmatrix} \frac{1}{\lambda_1-\lambda_2}\Big(\big(\gamma p_a - (\alpha+\lambda_2)(1-p_a)\big)e^{\lambda_1 t} + \big((\alpha+\lambda_1)(1-p_a) - \gamma p_a\big)e^{\lambda_2 t}\Big) \\ \frac{\alpha+\lambda_1}{\lambda_1-\lambda_2}\left(p_a - \frac{(\alpha+\lambda_2)(1-p_a)}{\gamma}\right)e^{\lambda_1 t} + \frac{\alpha+\lambda_2}{\lambda_1-\lambda_2}\left(\frac{(\alpha+\lambda_1)(1-p_a)}{\gamma} - p_a\right)e^{\lambda_2 t} \\ \frac{\delta_{\mathrm{c}}(\alpha+\lambda_1)}{\lambda_1(\lambda_1-\lambda_2)}\left(p_a - \frac{(\alpha+\lambda_2)(1-p_a)}{\gamma}\right)e^{\lambda_1 t} + \frac{\delta_{\mathrm{c}}(\alpha+\lambda_2)}{\lambda_2(\lambda_1-\lambda_2)}\left(\frac{(\alpha+\lambda_1)(1-p_a)}{\gamma} - p_a\right)e^{\lambda_2 t} + \frac{\delta_{\mathrm{c}}}{\lambda_1\lambda_2}\left(\alpha p_a - \frac{(\alpha+\lambda_1)(\alpha+\lambda_2)(1-p_a)}{\gamma}\right) \end{pmatrix}$$

$$\int_0^\infty \big(1 - F(t)\big) e^{\mathbf{W}t}\mathbf{x}_{\mathrm{a}} dt = \begin{pmatrix} \frac{\big(\gamma p_a - (\alpha+\lambda_2)(1-p_a)\big)}{\lambda_1-\lambda_2} K(\lambda_1) + \frac{\big((\alpha+\lambda_1)(1-p_a) - \gamma p_a\big)}{\lambda_1-\lambda_2} K(\lambda_2) \\ \frac{\alpha+\lambda_1}{\lambda_1-\lambda_2}\left(p_a - \frac{(\alpha+\lambda_2)(1-p_a)}{\gamma}\right)K(\lambda_1) + \frac{\alpha+\lambda_2}{\lambda_1-\lambda_2}\left(\frac{(\alpha+\lambda_1)(1-p_a)}{\gamma} - p_a\right)K(\lambda_2) \\ \frac{\delta_{\mathrm{c}}(\alpha+\lambda_1)}{\lambda_1(\lambda_1-\lambda_2)}\left(p_a - \frac{(\alpha+\lambda_2)(1-p_a)}{\gamma}\right)K(\lambda_1) + \frac{\delta_{\mathrm{c}}(\alpha+\lambda_2)}{\lambda_2(\lambda_1-\lambda_2)}\left(\frac{(\alpha+\lambda_1)(1-p_a)}{\gamma} - p_a\right)K(\lambda_2) + \frac{\delta_{\mathrm{c}}}{\lambda_1\lambda_2}\left(\alpha p_a - \frac{(\alpha+\lambda_1)(\alpha+\lambda_2)(1-p_a)}{\gamma}\right)\mu \end{pmatrix}$$

$$\mathbf{x}^* = \begin{pmatrix} S^* \\ C^* \\ C_D^* \end{pmatrix} = \begin{pmatrix} \frac{\big(\gamma p_a - (\alpha+\lambda_2)(1-p_a)\big)}{\lambda_1-\lambda_2}\frac{K(\lambda_1)}{\mu} + \frac{\big((\alpha+\lambda_1)(1-p_a) - \gamma p_a\big)}{\lambda_1-\lambda_2}\frac{K(\lambda_2)}{\mu} \\ \frac{\alpha+\lambda_1}{\lambda_1-\lambda_2}\left(p_a - \frac{(\alpha+\lambda_2)(1-p_a)}{\gamma}\right)\frac{K(\lambda_1)}{\mu} + \frac{\alpha+\lambda_2}{\lambda_1-\lambda_2}\left(\frac{(\alpha+\lambda_1)(1-p_a)}{\gamma} - p_a\right)\frac{K(\lambda_2)}{\mu} \\ \frac{\delta_{\mathrm{c}}(\alpha+\lambda_1)}{\lambda_1(\lambda_1-\lambda_2)}\left(p_a - \frac{(\alpha+\lambda_2)(1-p_a)}{\gamma}\right)\frac{K(\lambda_1)}{\mu} + \frac{\delta_{\mathrm{c}}(\alpha+\lambda_2)}{\lambda_2(\lambda_1-\lambda_2)}\left(\frac{(\alpha+\lambda_1)(1-p_a)}{\gamma} - p_a\right)\frac{K(\lambda_2)}{\mu} + \frac{\delta_{\mathrm{c}}}{\lambda_1\lambda_2}\left(\alpha p_a - \frac{(\alpha+\lambda_1)(\alpha+\lambda_2)(1-p_a)}{\gamma}\right) \end{pmatrix}$$

For the function K we require the moment generating function M of the mixed exponential–gamma distribution that was calibrated to the LTACH length of stay distribution as described above:

$$M(\lambda) = p_x(1 - \lambda/r_x)^{-1} + (1 - p_x)(1 - \lambda/r_g)^{-k}$$

Then,

$$K(\lambda) = \frac{M(\lambda) - 1}{\lambda}$$

Using the above formulas, we solved for the values of α and δ_c that produce an equilibrium result that meets the following two criteria:

1. The cross-sectional fraction of patients who are in one of the colonized states at equilibrium is the cross-sectional positivity f divided by the assumed surveillance test sensitivity σ:

$$C^* + C^*_{cd} = f/\sigma$$

2. The clinical detection rate in the facility at equilibrium is d:

$$\delta_c C^* = d$$

The values of f, σ, and d, as well as the other parameters aside from α and δ_c within the formulas for C^* and C^*_{cd} above are determined by the data and/or by assumption as described in Tables 1 and 2 in the main text. Therefore, we have two equations to solve simultaneously for the unique solution of α and δ_c.

Facility basic reproduction number formula derivation

For the general system defined in the main text, we will show that the facility basic reproduction number

$$R_0 = \frac{\boldsymbol{\beta}^{\mathrm{T}}\left(\int_0^\infty (1 - F(t)) e^{\mathbf{C}t} \int_0^t e^{-\mathbf{C}\tau} \mathbf{A} e^{\mathbf{S}\tau} \boldsymbol{\theta} d\tau\, dt\right)}{\mathbf{1}^{\mathrm{T}} \int_0^\infty (1 - F(t)) e^{\mathbf{S}t} \boldsymbol{\theta} dt},$$

where

$$\mathbf{S} = \begin{pmatrix} -s_{11} - \omega_1 & \cdots & s_{1n} \\ \vdots & \ddots & \vdots \\ s_{n1} & \cdots & -s_{nn} - \omega_n \end{pmatrix}, \quad \mathbf{C} = \begin{pmatrix} -c_{11} - \omega_{n+1} & \cdots & c_{1n} \\ \vdots & \ddots & \vdots \\ c_{n1} & \cdots & -c_{nn} - \omega_{n+m} \end{pmatrix}, \quad \mathbf{A} = \begin{pmatrix} a_{11} & \cdots & a_{1n} \\ \vdots & \ddots & \vdots \\ a_{m1} & \cdots & a_{mn} \end{pmatrix},$$

$$\boldsymbol{\beta} = \begin{pmatrix} \beta_1 \\ \vdots \\ \beta_m \end{pmatrix}, \quad \boldsymbol{\theta} = \begin{pmatrix} \theta_1 \\ \vdots \\ \theta_n \end{pmatrix}, \quad F(t) = 1 - e^{-\int_0^t h(\tau) d\tau}$$

To derive our equation we show that it is consistent with a "basic reproduction ratio" formula in Diekmann et al (6). Their definition depends on a measure of an individual's "h-state", η, just prior to acquiring colonization. First, they define the quantity $S(\eta)$: the distribution of h-states among susceptible individuals at equilibrium in the absence of any colonized patients. Then they define the quantity $A(\tau, \xi, \eta)$, which is the expected infectivity of an individual who was in h-state η just prior to acquisition time τ ago, toward a susceptible individual currently in h-state ξ.

In our model, the h-state is characterized by both the individual's susceptibility compartment, $s \in (1, \ldots, n)$, and time since admission, $t \in (0, \infty)$. So we have $S(\eta) = S(s_\eta, t_\eta)$ and $A(\tau, \xi, \eta) = A(\tau, s_\xi, t_\xi, s_\eta, t_\eta)$. The time-since-admission component is only relevant for the infectivity of the currently colonized individual represented by η, whose probability of removal (death or discharge) from the facility (thus no longer infective toward other facility patients) between t and $t + \tau$ might depend on t. However, the infectivity toward the susceptible individual represented by ξ depends only on that individual's current compartment s, via the susceptibility factor ψ_s (the

probability of being removed while susceptible will be incorporated in the S function). Therefore, A does not depend on t_ξ, and for simplicity of notation we let $t = t_\eta$ and seek expressions for the functions $S(s_\eta, t)$ and $A(\tau, s_\xi, s_\eta, t)$.

The infectivity A will be the susceptibility ψ_{s_ξ} times the sum of infectivity values β_c of each possible colonized compartment c, weighted by the probabilities $p(c, s_\eta, t, \tau)$ of being in colonized compartment c time τ after acquisition at stay-time t from compartment s_η and still in the facility. So we have:

$$A(\tau, s_\xi, s_\eta, t) = \psi_{s_\xi} \sum_{c=1}^{m} \beta_c p(c, \tau, s_\eta, t)$$

This expression for A satisfies a separability condition in Diekmann et al., i.e. it can be written as a product of two functions:

$$A(\tau, s_\xi, s_\eta, t) = a(s_\xi) B(\tau, s_\eta, t),$$

where

$$a(s_\xi) = \psi_{s_\xi}, \qquad B(\tau, s_\eta, t) = \sum_{c=1}^{m} \beta_c p(c, \tau, s_\eta, t)$$

Therefore, the following formula from Diekmann et al. for the basic reproduction ratio applies:

$$R_0 = \int_0^\infty \sum_{s_\eta=1}^{n} a(s_\eta) \int_0^\infty B(\tau, s_\eta, t) d\tau \, S(s_\eta, t) \, dt$$

For simplicity we let $s = s_\eta$ and specify the R_0 formula as

$$R_0 = \sum_{c=1}^{m} \beta_c \int_0^\infty \sum_{s=1}^{n} \int_0^\infty p(c, \tau, s, t) d\tau \, \psi_s S(s, t) \, dt$$

First, from our equilibrium results above we have that $S(s, t)$ is the sth element of the column vector:

$$\frac{(1 - F(t)) e^{\mathbf{S}t} \boldsymbol{\theta}}{\mathbf{1}^{\mathrm{T}} \int_0^\infty (1 - F(t)) e^{\mathbf{S}t} \boldsymbol{\theta} dt}$$

Next, we show that $p(c, \tau, s, t)$ is the element in the cth row, sth column of the matrix

$$(1 - F(t + \tau))/(1 - F(t)) \, e^{\mathbf{C}\tau} \mathbf{A}_0,$$

where $\mathbf{A}_0$ is the matrix $\mathbf{A}$ above with each column scaled by its sum. The sth column of $\mathbf{A}_0$ represents the distribution of colonized compartments initially entered when acquiring from compartment s, and $e^{\mathbf{C}\tau}$ projects the initial compartment forward τ time units according to the colonized state transition dynamics embedded in $\mathbf{C}$. The expression $(1 - F(t + \tau))/(1 - F(t))$ is the probability that removal does not occur before time-of-stay $t + \tau$ given that the stay was longer than t (the acquisition time).

So $\int_0^\infty p(c, \tau, s, t) d\tau$ is the element in the cth row, sth column of the matrix

$$\int_0^\infty \frac{1 - F(t + \tau)}{1 - F(t)} e^{\mathbf{C}\tau} d\tau \, \mathbf{A}_0$$

Noting that the sum of column s of the matrix $\mathbf{A}$ is ψ_s, we have that $\int_0^\infty p(c, \tau, s, t) d\tau \, \psi_s$ is the element in the cth row, sth column of the matrix

$$\int_0^\infty \frac{1 - F(t+\tau)}{1 - F(t)} e^{\mathbf{C}\tau} d\tau\, \mathbf{A}$$

Substituting into the R_0 formula above, we have

$$R_0 = \frac{\boldsymbol{\beta}^{\mathrm{T}} \int_0^\infty \left(\int_0^\infty \frac{1 - F(t+\tau)}{1 - F(t)} e^{\mathbf{C}\tau} d\tau \right) \mathbf{A} e^{\mathbf{S}t} \boldsymbol{\theta} \big(1 - F(t)\big) dt}{\mathbf{1}^{\mathrm{T}} \int_0^\infty \big(1 - F(t)\big) e^{\mathbf{S}t} \boldsymbol{\theta} dt}$$

$$R_0 = \frac{\boldsymbol{\beta}^{\mathrm{T}} \int_0^\infty \left(\int_0^\infty \big(1 - F(t+\tau)\big) e^{\mathbf{C}\tau} d\tau \right) \mathbf{A} e^{\mathbf{S}t} \boldsymbol{\theta} dt}{\mathbf{1}^{\mathrm{T}} \int_0^\infty \big(1 - F(t)\big) e^{\mathbf{S}t} \boldsymbol{\theta} dt}$$

We use the substitution $z = \tau + t$ and rearrange terms to get

$$R_0 = \frac{\boldsymbol{\beta}^{\mathrm{T}} \int_0^\infty \left(\int_t^\infty \big(1 - F(z)\big) e^{\mathbf{C}z} dz \right) e^{-\mathbf{C}t} \mathbf{A} e^{\mathbf{S}t} \boldsymbol{\theta} dt}{\mathbf{1}^{\mathrm{T}} \int_0^\infty \big(1 - F(t)\big) e^{\mathbf{S}t} \boldsymbol{\theta} dt}$$

Integrate the numerator by parts using the following substitutions:

$$u = \int_t^\infty \big(1 - F(z)\big) e^{\mathbf{C}z} dz, \quad v = \int_0^t e^{-\mathbf{C}\tau} \mathbf{A} e^{\mathbf{S}\tau} \boldsymbol{\theta} d\tau$$

$$du = -\big(1 - F(t)\big) e^{\mathbf{C}t} dt, \qquad dv = e^{-\mathbf{C}t} \mathbf{A} e^{\mathbf{S}t} \boldsymbol{\theta} dt$$

$[uv]_{t=0}^{t\to\infty} = 0$, so

$$R_0 = \frac{\boldsymbol{\beta}^{\mathrm{T}} \left(\int_0^\infty \big(1 - F(t)\big) e^{\mathbf{C}t} \int_0^t e^{-\mathbf{C}\tau} \mathbf{A} e^{\mathbf{S}\tau} \boldsymbol{\theta} d\tau\, dt \right)}{\mathbf{1}^{\mathrm{T}} \int_0^\infty \big(1 - F(t)\big) e^{\mathbf{S}t} \boldsymbol{\theta} dt}$$

In the numerator of this expression for R_0, the integral with respect to t can be calculated similarly to integral in the denominator, discussed above. I.e., if all state-dependent removal rates are nonzero, the elements of the vector $e^{\mathbf{C}t} \int_0^t e^{-\mathbf{C}\tau} \mathbf{A} e^{\mathbf{S}\tau} \boldsymbol{\theta} d\tau$ are linear combinations of $t^r e^{\lambda_i t}$ functions with negative eigenvalues λ_i of the $\mathbf{S}$ and $\mathbf{C}$ matrices and integer $r \geq 0$. Then, integrating the product of this vector and $\big(1 - F(t)\big)$ produces a vector with linear combinations of $K^{(r)}(\lambda_i)$, where $K(x) = \big(1 - M(x)\big)/x$ and $M(x)$ is the moment generating function of the distribution for which F is the cumulative distribution function. If there are state-dependent removal rates equal to zero, then the vector $e^{\mathbf{C}t} \int_0^t e^{-\mathbf{C}\tau} \mathbf{A} e^{\mathbf{S}\tau} \boldsymbol{\theta} d\tau$ may contain a term linear in t. In which case we evaluate

$$\int_0^\infty t\big(1 - F(t)\big) dt = \left[\frac{t^2}{2} \big(1 - F(t)\big) \right]_0^\infty + \frac{1}{2} \int_0^\infty t^2 f(t) dt = (0 - 0) + \frac{1}{2}(\mu^2 + \sigma^2) = \frac{\mu^2 + \sigma^2}{2},$$

where μ and σ are the mean and standard deviation of the distribution for which F is the cumulative distribution function.

Examples:

No time-dependent removal hazard

For the model with removal hazard $h(t) = 0$, and thus $F(t) = 0$, it is convenient to return to the following expression for R_0 within the derivation above:

$$R_0 = \frac{\boldsymbol{\beta}^{\mathrm{T}} \int_0^\infty \left(\int_0^\infty \big(1 - F(t+\tau)\big) e^{\mathbf{C}\tau} d\tau \right) \mathbf{A} e^{\mathbf{S}t} \boldsymbol{\theta} dt}{\mathbf{1}^{\mathrm{T}} \int_0^\infty \big(1 - F(t)\big) e^{\mathbf{S}t} \boldsymbol{\theta} dt}$$

Plugging in $F(t) = 0$, we have

$$R_0 = \frac{\boldsymbol{\beta}^{\mathrm{T}} \int_0^\infty \left(\int_0^\infty e^{\mathbf{C}\tau} d\tau \right) \mathbf{A} e^{\mathbf{S}t} \boldsymbol{\theta} dt}{\mathbf{1}^{\mathrm{T}} \int_0^\infty e^{\mathbf{S}t} \boldsymbol{\theta} dt}$$

The following are true for invertible matrices $\mathbf{C}$ and $\mathbf{S}$:

$$\int_0^\infty e^{\mathbf{C}\tau} d\tau = -\mathbf{C}^{-1}, \qquad \int_0^\infty e^{\mathbf{S}t} dt = -\mathbf{S}^{-1},$$

hence

$$R_0 = -\boldsymbol{\beta}^{\mathrm{T}} \mathbf{C}^{-1} \mathbf{A} \left(\frac{\mathbf{S}^{-1} \boldsymbol{\theta}}{\mathbf{1}^{\mathrm{T}} \mathbf{S}^{-1} \boldsymbol{\theta}} \right)$$

Model 1: Simple susceptible–colonized model

$$\frac{dS}{dt} = -\left(\alpha + h(t)\right) S$$

$$\frac{dC}{dt} = \alpha S - h(t) C$$

$$\alpha = \beta C$$

$$\mathbf{S} = 0, \qquad \mathbf{C} = 0, \qquad \mathbf{A} = 1, \qquad \boldsymbol{\theta} = 1, \qquad \boldsymbol{\beta} = \beta$$

$$R_0 = \frac{\boldsymbol{\beta}^{\mathrm{T}} \left(\int_0^\infty \left(1 - F(t)\right) e^{\mathbf{C}t} \int_0^t e^{-\mathbf{C}\tau} \mathbf{A} e^{\mathbf{S}\tau} \boldsymbol{\theta} d\tau \, dt \right)}{\mathbf{1}^{\mathrm{T}} \int_0^\infty \left(1 - F(t)\right) e^{\mathbf{S}t} \boldsymbol{\theta} dt} = \frac{\beta \left(\int_0^\infty \left(1 - F(t)\right) \int_0^t d\tau \, dt \right)}{\int_0^\infty \left(1 - F(t)\right) dt} = \frac{\beta \left(\int_0^\infty \left(1 - F(t)\right) t dt \right)}{\int_0^\infty \left(1 - F(t)\right) dt}$$

$$R_0 = \beta \left(\frac{\mu^2 + \sigma^2}{2\mu} \right)$$

Model 2: Clearance of colonization

$$\frac{dS}{dt} = -\left(\alpha + h(t)\right) S + \gamma C$$

$$\frac{dC}{dt} = \alpha S - \left(\gamma + h(t)\right) C$$

$$\alpha = \beta C$$

$$\mathbf{S} = -\omega, \qquad \mathbf{C} = -\gamma, \qquad \mathbf{A} = 1, \qquad \boldsymbol{\theta} = 1, \qquad \boldsymbol{\beta} = \beta$$

$$R_0 = \frac{\boldsymbol{\beta}^{\mathrm{T}} \left(\int_0^\infty \left(1 - F(t)\right) e^{\mathbf{C}t} \int_0^t e^{-\mathbf{C}\tau} \mathbf{A} e^{\mathbf{S}\tau} \boldsymbol{\theta} d\tau \, dt \right)}{\mathbf{1}^{\mathrm{T}} \int_0^\infty \left(1 - F(t)\right) e^{\mathbf{S}t} \boldsymbol{\theta} dt} = \frac{\beta \left(\int_0^\infty \left(1 - F(t)\right) e^{-\gamma t} \int_0^t e^{\gamma \tau} d\tau \, dt \right)}{\int_0^\infty \left(1 - F(t)\right) dt}$$

$$= \frac{\beta \left(\int_0^\infty \left(1 - F(t)\right) dt - \int_0^\infty \left(1 - F(t)\right) e^{-\gamma t} dt \right)}{\gamma \int_0^\infty \left(1 - F(t)\right) dt} = \frac{\beta \left(\mu - K(-\gamma)\right)}{\gamma \mu}$$

$$R_0 = \frac{\beta}{\gamma} \left(1 - \frac{K(-\gamma)}{\mu} \right)$$

With length of stay exponentially distributed with rate r:

$$K(\lambda) = (M(\lambda) - 1)/\lambda = ((1 - \lambda/r)^{-1} - 1)/\lambda = 1/(r - \lambda)$$

$$K(-\gamma) = 1/(r + \gamma)\,, \qquad \mu = 1/r$$

$$R_0^{\text{exp}} = \frac{\beta}{\gamma}\left(1 - \frac{1}{\mu(r+\gamma)}\right) = \frac{\beta}{\gamma}\left(1 - \frac{r}{r+\gamma}\right) = \frac{\beta}{\gamma}\left(\frac{\gamma}{r+\gamma}\right) = \beta\left(\frac{1}{r+\gamma}\right)$$

With length of stay gamma distributed with rate parameter r and shape parameter k

$$K(\lambda) = (M(\lambda) - 1)/\lambda = ((1 - \lambda/r)^{-k} - 1)/\lambda$$

$$K(-\gamma) = (1 - (1 + \gamma/r)^{-k})/\gamma\,, \qquad \mu = k/r$$

$$R_0^{\text{gam}} = \frac{\beta}{\gamma}\left(1 - \frac{1 - (1+\gamma/r)^{-k}}{k\gamma/r}\right)$$

Model 3: Clinical detection with contact precautions

$$\frac{dS}{dt} = -\big(\alpha + h(t)\big)S + \gamma C$$

$$\frac{dC}{dt} = \alpha S - \big(\delta_{\text{c}} + \gamma + h(t)\big)C$$

$$\frac{dC_{\text{cd}}}{dt} = \delta_{\text{c}} C - h(t) C_{\text{cd}}$$

$$\alpha = \beta(C + (1-\varepsilon)C_{\text{cd}})$$

$$\mathbf{S} = 0, \qquad \mathbf{C} = \begin{pmatrix} -\delta_{\text{c}} - \gamma & 0 \\ \delta_{\text{c}} & 0 \end{pmatrix}, \qquad \mathbf{A} = \begin{pmatrix} 1 \\ 0 \end{pmatrix}, \qquad \boldsymbol{\theta} = 1, \qquad \boldsymbol{\beta} = \begin{pmatrix} \beta \\ \beta(1-\varepsilon) \end{pmatrix}$$

$$R_0 = \frac{\boldsymbol{\beta}^{\text{T}}\left(\int_0^\infty \big(1 - F(t)\big) e^{\mathbf{C}t} \int_0^t e^{-\mathbf{C}\tau} \mathbf{A} e^{\mathbf{S}\tau} \boldsymbol{\theta}\, d\tau\, dt\right)}{\mathbf{1}^{\text{T}} \int_0^\infty \big(1 - F(t)\big) e^{\mathbf{S}t} \boldsymbol{\theta}\, dt}$$

$$e^{\mathbf{C}t} = \begin{pmatrix} e^{-(\delta_{\text{c}}+\gamma)t} & 0 \\ \dfrac{\delta_{\text{c}}}{\delta_{\text{c}}+\gamma}\left(e^{-\omega t} - e^{-(\delta_{\text{c}}+\gamma)t}\right) & 1 \end{pmatrix}$$

$$\int_0^t e^{-\mathbf{C}\tau} \mathbf{A} e^{\mathbf{S}\tau} \boldsymbol{\theta}\, d\tau = \int_0^t \begin{pmatrix} e^{(\delta_{\text{c}}+\gamma)\tau} & 0 \\ \dfrac{\delta_{\text{c}}}{\delta_{\text{c}}+\gamma}\left(1 - e^{(\delta_{\text{c}}+\gamma)\tau}\right) & 1 \end{pmatrix} \begin{pmatrix} 1 \\ 0 \end{pmatrix} d\tau = \int_0^t \begin{pmatrix} e^{(\delta_{\text{c}}+\gamma)\tau} \\ \dfrac{\delta_{\text{c}}}{\delta_{\text{c}}+\gamma}\left(1 - e^{(\delta_{\text{c}}+\gamma)\tau}\right) \end{pmatrix} d\tau$$
$$= \begin{pmatrix} \dfrac{1}{\delta_{\text{c}}+\gamma}\left(e^{(\delta_{\text{c}}+\gamma)t} - 1\right) \\ \dfrac{\delta_{\text{c}}}{\delta_{\text{c}}+\gamma}\left(t - \dfrac{1}{\delta_{\text{c}}+\gamma}\left(e^{(\delta_{\text{c}}+\gamma)t} - 1\right)\right) \end{pmatrix}$$

$$e^{\mathbf{C}t}\int_0^t e^{-\mathbf{C}\tau}\mathbf{A}e^{\mathbf{S}\tau}\boldsymbol{\theta}d\tau = \begin{pmatrix} e^{-(\delta_c+\gamma)t} & 0 \\ \frac{\delta_c}{\delta_c+\gamma}\left(e^{-\omega t}-e^{-(\delta_c+\gamma)t}\right) & e^{-\omega t}\end{pmatrix}\begin{pmatrix} \frac{1}{\delta_c+\gamma}\left(e^{(\delta_c+\gamma)t}-1\right) \\ \frac{\delta_c}{\delta_c+\gamma}\left(t-\frac{1}{\delta_c+\gamma}\left(e^{(\delta_c+\gamma)t}-1\right)\right)\end{pmatrix}$$

$$= \begin{pmatrix} \frac{1}{\delta_c+\gamma}\left(1-e^{-(\delta_c+\gamma)t}\right) \\ \frac{\delta_c}{\delta_c+\gamma}t-\frac{\delta_c}{(\delta_c+\gamma)^2}\left(1-e^{-(\delta_c+\gamma)t}\right)\end{pmatrix}$$

$$\int_0^\infty (1-F(t))e^{\mathbf{C}t}\int_0^t e^{-\mathbf{C}\tau}\mathbf{A}e^{\mathbf{S}\tau}d\tau\, dt = \begin{pmatrix} \frac{1}{\delta_c+\gamma}\left(\mu-K(-\delta_c-\gamma)\right) \\ \frac{\delta_c}{\delta_c+\gamma}\left(\frac{\mu^2+\sigma^2}{2}\right)-\frac{\delta_c}{(\delta_c+\gamma)^2}\left(\mu-K(-\delta_c-\gamma)\right)\end{pmatrix}$$

$$R_0 = \begin{pmatrix}\beta \\ \beta(1-\varepsilon)\end{pmatrix}^{\mathrm{T}}\begin{pmatrix} \frac{1}{\delta_c+\gamma}\left(\mu-K(-\delta_c-\gamma)\right) \\ \frac{\delta_c}{\delta_c+\gamma}\left(\frac{\mu^2+\sigma^2}{2}\right)-\frac{\delta_c}{(\delta_c+\gamma)^2}\left(\mu-K(-\delta_c-\gamma)\right)\end{pmatrix}\frac{1}{\mu}$$

$$R_0 = \frac{\beta}{\delta_c+\gamma}\left(1-\frac{K(-\delta_c-\gamma)}{\mu}+(1-\varepsilon)\left(\delta_c\frac{\mu^2+\sigma^2}{2\mu}-\frac{\delta_c}{\delta_c+\gamma}\left(1-\frac{K(-\delta_c-\gamma)}{\mu}\right)\right)\right)$$

$$R_0 = \frac{\beta}{\delta_c+\gamma}\left(\left(1-\frac{\delta_c(1-\varepsilon)}{\delta_c+\gamma}\right)\left(1-\frac{K(-\delta_c-\gamma)}{\mu}\right)+\delta_c(1-\varepsilon)\frac{\mu^2+\sigma^2}{2\mu}\right)$$

$$K(x) = \frac{M(x)-1}{x}$$

When applying this model to LTACH data, we assume a mixed exponential-gamma distribution for the length of stay, with a portion p_x of patients following an exponential distribution with mean μ_x and the rest following a gamma distribution with mean μ_g and shape parameter k. For this distribution:

$$M(x) = p_x(1-\mu_x x)^{-1}+(1-p_x)\left(1-\mu_g x/k\right)^{-k}$$

Model 4: Active surveillance and decolonization

$$\frac{dS}{dt} = -\left(\alpha+h(t)\right)S+\gamma C$$

$$\frac{dS_{\mathrm{sd}}}{dt} = -\left((1-\varepsilon)\alpha+h(t)\right)S_{\mathrm{sd}}+\gamma_{\mathrm{d}}C_{\mathrm{sd}}$$

$$\frac{dC}{dt} = \alpha S-\left(\delta_s+\delta_c+\gamma+h(t)\right)C$$

$$\frac{dC_{\mathrm{sd}}}{dt} = (1-\varepsilon)\alpha S_{\mathrm{sd}}+\delta_s C-\left(\delta_c+\gamma_{\mathrm{d}}+h(t)\right)C_{\mathrm{sd}}$$

$$\frac{dC_{\mathrm{cd}}}{dt} = \delta_c C+\delta_c C_{\mathrm{sd}}-h(t)C_{\mathrm{cd}}$$

$$\alpha = \beta\left(C+(1-\varepsilon)(C_{\mathrm{sd}}+C_{\mathrm{cd}})\right)$$

$$\mathbf{S} = \begin{pmatrix} 0 & 0 \\ 0 & 0 \end{pmatrix}, \qquad \mathbf{C} = \begin{pmatrix} -\delta_\mathrm{s} - \delta_\mathrm{c} - \gamma & 0 & 0 \\ \delta_\mathrm{s} & -\delta_\mathrm{c} - \gamma_\mathrm{d} & 0 \\ \delta_\mathrm{c} & \delta_\mathrm{c} & 0 \end{pmatrix}$$

$$\mathbf{A} = \begin{pmatrix} 1 & 0 \\ 0 & 1-\varepsilon \\ 0 & 0 \end{pmatrix}, \qquad \boldsymbol{\theta} = \begin{pmatrix} 1 \\ 0 \end{pmatrix}, \qquad \boldsymbol{\beta} = \begin{pmatrix} \beta \\ \beta(1-\varepsilon) \\ \beta(1-\varepsilon) \end{pmatrix}$$

$$\mathbf{A}e^{\mathbf{S}\tau}\boldsymbol{\theta} = \begin{pmatrix} 1 & 0 \\ 0 & 1-\varepsilon \\ 0 & 0 \end{pmatrix} \begin{pmatrix} 1 & 0 \\ 0 & 1 \end{pmatrix} \begin{pmatrix} 1 \\ 0 \end{pmatrix} = \begin{pmatrix} 1 \\ 0 \\ 0 \end{pmatrix}$$

When $\delta_\mathrm{s} - (\gamma_\mathrm{d} - \gamma) \neq 0$:

$$e^{\mathbf{C}t} = \begin{pmatrix} e^{-(\delta_\mathrm{s}+\delta_\mathrm{c}+\gamma)t} & 0 & 0 \\ \frac{\delta_\mathrm{s}}{\delta_\mathrm{s} - (\gamma_\mathrm{d} - \gamma)}\left(e^{-(\delta_\mathrm{c}+\gamma_\mathrm{d})t} - e^{-(\delta_\mathrm{s}+\delta_\mathrm{c}+\gamma)t}\right) & e^{-(\delta_\mathrm{c}+\gamma_\mathrm{d})t} & 0 \\ \frac{\delta_\mathrm{c}}{\delta_\mathrm{s} - (\gamma_\mathrm{d} - \gamma)}\left(\frac{\delta_\mathrm{s}}{\delta_\mathrm{c}+\gamma_\mathrm{d}}\left(1 - e^{-(\delta_\mathrm{c}+\gamma_\mathrm{d})t}\right) - \frac{\gamma_\mathrm{d} - \gamma}{\delta_\mathrm{s}+\delta_\mathrm{c}+\gamma}\left(1 - e^{-(\delta_\mathrm{s}+\delta_\mathrm{c}+\gamma)t}\right)\right) & \frac{\delta_\mathrm{c}}{\delta_\mathrm{c}+\gamma_\mathrm{d}}\left(1 - e^{-(\delta_\mathrm{c}+\gamma_\mathrm{d})t}\right) & 1 \end{pmatrix}$$

$$e^{-\mathbf{C}\tau}\mathbf{A}e^{\mathbf{S}\tau}\boldsymbol{\theta} = \begin{pmatrix} e^{(\delta_\mathrm{s}+\delta_\mathrm{c}+\gamma)\tau} \\ \frac{\delta_\mathrm{s}}{\delta_\mathrm{s} - (\gamma_\mathrm{d} - \gamma)}\left(e^{(\delta_\mathrm{c}+\gamma_\mathrm{d})\tau} - e^{(\delta_\mathrm{s}+\delta_\mathrm{c}+\gamma)\tau}\right) \\ \frac{\delta_\mathrm{c}}{\delta_\mathrm{s} - (\gamma_\mathrm{d} - \gamma)}\left(\frac{\delta_\mathrm{s}}{\delta_\mathrm{c}+\gamma_\mathrm{d}}\left(1 - e^{(\delta_\mathrm{c}+\gamma_\mathrm{d})\tau}\right) - \frac{\gamma_\mathrm{d} - \gamma}{\delta_\mathrm{s}+\delta_\mathrm{c}+\gamma}\left(1 - e^{(\delta_\mathrm{s}+\delta_\mathrm{c}+\gamma)\tau}\right)\right) \end{pmatrix}$$

$$\int_0^t e^{-\mathbf{C}\tau}\mathbf{A}e^{\mathbf{S}\tau}\boldsymbol{\theta}\,d\tau = \begin{pmatrix} \frac{1}{\delta_\mathrm{s}+\delta_\mathrm{c}+\gamma}\left(e^{(\delta_\mathrm{s}+\delta_\mathrm{c}+\gamma)t} - 1\right) \\ \frac{\delta_\mathrm{s}}{\delta_\mathrm{s} - (\gamma_\mathrm{d} - \gamma)}\left(\frac{1}{\delta_\mathrm{c}+\gamma_\mathrm{d}}\left(e^{(\delta_\mathrm{c}+\gamma_\mathrm{d})t} - 1\right) - \frac{1}{\delta_\mathrm{s}+\delta_\mathrm{c}+\gamma}\left(e^{(\delta_\mathrm{s}+\delta_\mathrm{c}+\gamma)t} - 1\right)\right) \\ \frac{\delta_\mathrm{c}}{\delta_\mathrm{s} - (\gamma_\mathrm{d} - \gamma)}\left(\frac{\delta_\mathrm{s}}{\delta_\mathrm{c}+\gamma_\mathrm{d}}\left(t - \frac{1}{\delta_\mathrm{c}+\gamma_\mathrm{d}}\left(e^{(\delta_\mathrm{c}+\gamma_\mathrm{d})t} - 1\right)\right) - \frac{\gamma_\mathrm{d} - \gamma}{\delta_\mathrm{s}+\delta_\mathrm{c}+\gamma}\left(t - \frac{1}{\delta_\mathrm{s}+\delta_\mathrm{c}+\gamma}\left(e^{(\delta_\mathrm{s}+\delta_\mathrm{c}+\gamma)\tau} - 1\right)\right)\right) \end{pmatrix}$$

$$e^{\mathbf{C}t}\int_0^t e^{-\mathbf{C}\tau}\mathbf{A}e^{\mathbf{S}\tau}\boldsymbol{\theta}\,d\tau = \begin{pmatrix} \frac{1}{\delta_\mathrm{s}+\delta_\mathrm{c}+\gamma}\left(1 - e^{-(\delta_\mathrm{s}+\delta_\mathrm{c}+\gamma)t}\right) \\ \frac{\delta_\mathrm{s}}{\delta_\mathrm{s} - (\gamma_\mathrm{d} - \gamma)}\left(\frac{1}{\delta_\mathrm{c}+\gamma_\mathrm{d}}\left(1 - e^{-(\delta_\mathrm{c}+\gamma_\mathrm{d})t}\right) - \frac{1}{\delta_\mathrm{s}+\delta_\mathrm{c}+\gamma}\left(1 - e^{-(\delta_\mathrm{s}+\delta_\mathrm{c}+\gamma)t}\right)\right) \\ \frac{\delta_\mathrm{c}}{\delta_\mathrm{s} - (\gamma_\mathrm{d} - \gamma)}\left(\left(\frac{\delta_\mathrm{s}}{\delta_\mathrm{c}+\gamma_\mathrm{d}} - \frac{\gamma_\mathrm{d} - \gamma}{\delta_\mathrm{s}+\delta_\mathrm{c}+\gamma}\right)t + \frac{\gamma_\mathrm{d} - \gamma}{(\delta_\mathrm{s}+\delta_\mathrm{c}+\gamma)^2}\left(1 - e^{-(\delta_\mathrm{s}+\delta_\mathrm{c}+\gamma)t}\right) - \frac{\delta_\mathrm{s}}{(\delta_\mathrm{c}+\gamma_\mathrm{d})^2}\left(1 - e^{-(\delta_\mathrm{c}+\gamma_\mathrm{d})t}\right)\right) \end{pmatrix}$$

$$\frac{\int_0^\infty (1-F(t))e^{\mathbf{C}t}\int_0^t e^{-\mathbf{C}\tau}\mathbf{A}e^{\mathbf{S}\tau}\boldsymbol{\theta}d\tau\,dt}{\mathbf{1}^{\mathrm{T}}\int_0^\infty (1-F(t))e^{\mathbf{S}t}\boldsymbol{\theta}dt}$$

$$= \begin{pmatrix} \frac{1}{\delta_s+\delta_c+\gamma}\left(1-\frac{K(-\delta_s-\delta_c-\gamma)}{\mu}\right) \\ \frac{\delta_s}{\delta_s-(\gamma_d-\gamma)}\left(\frac{1}{\delta_c+\gamma_d}\left(1-\frac{K(-\delta_c-\gamma)}{\mu}\right)-\frac{1}{\delta_s+\delta_c+\gamma}\left(1-\frac{K(-\delta_s-\delta_c-\gamma)}{\mu}\right)\right) \\ \frac{\delta_c}{\delta_s-(\gamma_d-\gamma)}\left(\left(\frac{\delta_s}{\delta_c+\gamma_d}-\frac{\gamma_d-\gamma}{\delta_s+\delta_c+\gamma}\right)\frac{\mu^2+\sigma^2}{\mu}+\frac{\gamma_d-\gamma}{(\delta_s+\delta_c+\gamma)^2}\left(1-\frac{K(-\delta_s-\delta_c-\gamma)}{\mu}\right)-\frac{\delta_s}{(\delta_c+\gamma_d)^2}\left(1-\frac{K(-\delta_c-\gamma_d)}{\mu}\right)\right) \end{pmatrix}$$

$$R_0 = \frac{\boldsymbol{\beta}^{\mathrm{T}}\left(\int_0^\infty (1-F(t))e^{\mathbf{C}t}\int_0^t e^{-\mathbf{C}\tau}\mathbf{A}e^{\mathbf{S}\tau}\boldsymbol{\theta}d\tau\,dt\right)}{\mathbf{1}^{\mathrm{T}}\int_0^\infty (1-F(t))e^{\mathbf{S}t}\boldsymbol{\theta}dt}$$

$$R_0 = \frac{\beta}{\delta_s+\delta_c+\gamma}\left(1+\frac{1-\varepsilon}{\delta_s-(\gamma_d-\gamma)}\left(\frac{\delta_c(\gamma_d-\gamma)}{\delta_s+\delta_c+\gamma}-\delta_s\right)\right)\left(1-\frac{K(-\delta_s-\delta_c-\gamma)}{\mu}\right) + \frac{\beta(1-\varepsilon)}{\delta_s-(\gamma_d-\gamma)}\left(\frac{\delta_s\gamma_d}{(\delta_c+\gamma_d)^2}\left(1-\frac{K(-\delta_c-\gamma_d)}{\mu}\right)+\left(\frac{\delta_c\delta_s}{\delta_c+\gamma_d}-\frac{\delta_c(\gamma_d-\gamma)}{\delta_s+\delta_c+\gamma}\right)\frac{\mu^2+\sigma^2}{2\mu}\right)$$

When $\delta_s-(\gamma_d-\gamma)=0$:

$$e^{\mathbf{C}t} = \begin{pmatrix} e^{-(\delta_s+\delta_c+\gamma)t} & 0 & 0 \\ \delta_s t e^{-(\delta_s+\delta_c+\gamma)t} & e^{-(\delta_s+\delta_c+\gamma)t} & 0 \\ \frac{\delta_c}{\delta_s+\delta_c+\gamma}\left(\left(1+\frac{\delta_s}{\delta_s+\delta_c+\gamma}\right)\left(1-e^{-(\delta_s+\delta_c+\gamma)t}\right)-\delta_s t e^{-(\delta_s+\delta_c+\gamma)t}\right) & \frac{\delta_c}{\delta_s+\delta_c+\gamma}\left(1-e^{-(\delta_s+\delta_c+\gamma)t}\right) & 1 \end{pmatrix}$$

$$e^{-\mathbf{C}\tau}\mathbf{A}e^{\mathbf{S}\tau}\boldsymbol{\theta} = \begin{pmatrix} e^{(\delta_s+\delta_c+\gamma)\tau} \\ -\delta_s\tau e^{(\delta_s+\delta_c+\gamma)\tau} \\ \frac{\delta_c}{\delta_s+\delta_c+\gamma}\left(\left(1+\frac{\delta_s}{\delta_s+\delta_c+\gamma}\right)\left(1-e^{(\delta_s+\delta_c+\gamma)\tau}\right)+\delta_s\tau e^{(\delta_s+\delta_c+\gamma)\tau}\right) \end{pmatrix}$$

$$\int_0^t e^{-\mathbf{C}\tau}\mathbf{A}e^{\mathbf{S}\tau}\boldsymbol{\theta}d\tau = \begin{pmatrix} \frac{1}{\delta_s+\delta_c+\gamma}\left(e^{(\delta_s+\delta_c+\gamma)t}-1\right) \\ \frac{\delta_s}{(\delta_s+\delta_c+\gamma)^2}\left(e^{(\delta_s+\delta_c+\gamma)t}-1\right)-\frac{\delta_s}{\delta_s+\delta_c+\gamma}te^{(\delta_s+\delta_c+\gamma)t} \\ \frac{\delta_c}{\delta_s+\delta_c+\gamma}\left(\left(1+\frac{\delta_s}{\delta_s+\delta_c+\gamma}\right)\left(t-\frac{1}{\delta_s+\delta_c+\gamma}\left(e^{(\delta_s+\delta_c+\gamma)t}-1\right)\right)+\frac{\delta_s}{\delta_s+\delta_c+\gamma}te^{(\delta_s+\delta_c+\gamma)t}-\frac{\delta_s}{(\delta_s+\delta_c+\gamma)^2}\left(e^{(\delta_s+\delta_c+\gamma)t}-1\right)\right) \end{pmatrix}$$

$$e^{\mathbf{C}t}\int_0^t e^{-\mathbf{C}\tau}\mathbf{A}e^{\mathbf{S}\tau}\boldsymbol{\theta}d\tau = \begin{pmatrix} \frac{1}{\delta_s+\delta_c+\gamma}\left(1-e^{-(\delta_s+\delta_c+\gamma)t}\right) \\ \frac{\delta_s}{(\delta_s+\delta_c+\gamma)^2}\left(1-e^{-(\delta_s+\delta_c+\gamma)t}\right)-\frac{\delta_s}{\delta_s+\delta_c+\gamma}te^{-(\delta_s+\delta_c+\gamma)t} \\ \frac{\delta_c}{\delta_s+\delta_c+\gamma}\left(\frac{\delta_s}{\delta_s+\delta_c+\gamma}te^{-(\delta_s+\delta_c+\gamma)t}-\left(\frac{1}{\delta_s+\delta_c+\gamma}+\frac{2\delta_s}{(\delta_s+\delta_c+\gamma)^2}\right)\left(1-e^{-(\delta_s+\delta_c+\gamma)t}\right)+\left(1+\frac{\delta_s}{\delta_s+\delta_c+\gamma}\right)t\right) \end{pmatrix}$$

$$\frac{\int_0^\infty \left(1-F(t)\right)e^{\mathbf{C}t}\int_0^t e^{-\mathbf{C}\tau}\mathbf{A}e^{\mathbf{S}\tau}\boldsymbol{\theta}\,d\tau\,dt}{\mathbf{1}^{\mathrm{T}}\int_0^\infty \left(1-F(t)\right)e^{\mathbf{S}t}\boldsymbol{\theta}\,dt}$$
$$= \begin{pmatrix} \frac{1}{\delta_{\mathrm{s}}+\delta_{\mathrm{c}}+\gamma}\left(1-\frac{K(-\delta_{\mathrm{s}}-\delta_{\mathrm{c}}-\gamma)}{\mu}\right) \\ \frac{\delta_{\mathrm{s}}}{(\delta_{\mathrm{s}}+\delta_{\mathrm{c}}+\gamma)^2}\left(1-\frac{K(-\delta_{\mathrm{s}}-\delta_{\mathrm{c}}-\gamma)}{\mu}\right)-\frac{\delta_{\mathrm{s}}}{\delta_{\mathrm{s}}+\delta_{\mathrm{c}}+\gamma}\frac{K'(-\delta_{\mathrm{s}}-\delta_{\mathrm{c}}-\gamma)}{\mu} \\ \frac{\delta_{\mathrm{c}}}{\delta_{\mathrm{s}}+\delta_{\mathrm{c}}+\gamma}\left(\frac{\delta_{\mathrm{s}}}{\delta_{\mathrm{s}}+\delta_{\mathrm{c}}+\gamma}\frac{K'(-\delta_{\mathrm{s}}-\delta_{\mathrm{c}}-\gamma)}{\mu}-\left(\frac{1}{\delta_{\mathrm{s}}+\delta_{\mathrm{c}}+\gamma}+\frac{2\delta_{\mathrm{s}}}{(\delta_{\mathrm{s}}+\delta_{\mathrm{c}}+\gamma)^2}\right)\left(1-\frac{K(-\delta_{\mathrm{s}}-\delta_{\mathrm{c}}-\gamma)}{\mu}\right)+\left(1+\frac{\delta_{\mathrm{s}}}{\delta_{\mathrm{s}}+\delta_{\mathrm{c}}+\gamma}\right)\frac{\mu^2+\sigma^2}{\mu}\right) \end{pmatrix}$$

$$R_0 = \frac{\boldsymbol{\beta}^{\mathrm{T}}\left(\int_0^\infty \left(1-F(t)\right)e^{\mathbf{C}t}\int_0^t e^{-\mathbf{C}\tau}\mathbf{A}e^{\mathbf{S}\tau}\boldsymbol{\theta}\,d\tau\,dt\right)}{\mathbf{1}^{\mathrm{T}}\int_0^\infty \left(1-F(t)\right)e^{\mathbf{S}t}\boldsymbol{\theta}\,dt}$$

$$R_0 = \frac{\beta}{\delta_{\mathrm{s}}+\delta_{\mathrm{c}}+\gamma}\left(1+\frac{(1-\varepsilon)}{\delta_{\mathrm{s}}+\delta_{\mathrm{c}}+\gamma}\left(\delta_{\mathrm{s}}-\delta_{\mathrm{c}}-\frac{2\delta_{\mathrm{s}}\delta_{\mathrm{c}}}{\delta_{\mathrm{s}}+\delta_{\mathrm{c}}+\gamma}\right)\right)\left(1-\frac{K(-\delta_{\mathrm{s}}-\delta_{\mathrm{c}}-\gamma)}{\mu}\right) + \beta(1-\varepsilon)\left(\left(\frac{\delta_{\mathrm{s}}\delta_{\mathrm{c}}}{\delta_{\mathrm{s}}+\delta_{\mathrm{c}}+\gamma}-\delta_{\mathrm{s}}\right)\frac{K'(-\delta_{\mathrm{s}}-\delta_{\mathrm{c}}-\gamma)}{\mu}+\left(\delta_{\mathrm{c}}+\frac{\delta_{\mathrm{s}}\delta_{\mathrm{c}}}{\delta_{\mathrm{s}}+\delta_{\mathrm{c}}+\gamma}\right)\frac{\mu^2+\sigma^2}{\mu}\right)$$

Numerical calculation of R_0

As demonstrated in the examples above, when the matrix exponentials $e^{\mathbf{S}t}$ and $e^{\mathbf{C}t}$ can be expressed symbolically for a particular model, the R_0 formula we derived can be used directly to find a formula for R_0 in terms of the model parameters. We can also calculate R_0 numerically using the following procedure.

First, we create the following matrix **M**:

$$\mathbf{M} = \begin{pmatrix} \mathbf{S} & \mathbf{0} \\ \mathbf{A} & \mathbf{C} \end{pmatrix}, \qquad \boldsymbol{\theta}_{\mathbf{M}} = \begin{pmatrix} \boldsymbol{\theta} \\ \mathbf{0} \end{pmatrix}$$

With $\mathbf{S}$ and $\mathbf{C}$ being $n \times n$ and $m \times m$ matrices, respectively, the $\mathbf{0}$ in the upper-right corner of **M** represents an $n \times m$ block of zeros. The $\mathbf{0}$ in the column vector $\boldsymbol{\theta}_{\mathbf{M}}$ represents a column vector of *m* zeros.

The system

$$\frac{d\mathbf{x}}{dt} = \mathbf{M}\mathbf{x}, \qquad \mathbf{x}(0) = \boldsymbol{\theta}_{\mathbf{M}}$$

can be decoupled into two linked systems:

$$\frac{d\mathbf{x}_{\mathrm{s}}}{dt} = \mathbf{S}\mathbf{x}_{\mathrm{s}}, \qquad \mathbf{x}_{\mathrm{s}}(0) = \boldsymbol{\theta}$$

$$\frac{d\mathbf{x}_{\mathrm{c}}}{dt} = \mathbf{C}\mathbf{x}_{\mathrm{c}} + \mathbf{A}\mathbf{x}_{\mathrm{s}}, \qquad \mathbf{x}_{\mathrm{c}}(0) = \mathbf{0}$$

Solved sequentially we get:

$$\mathbf{x}_{\mathrm{s}}(t) = e^{\mathbf{S}t}\boldsymbol{\theta}, \qquad \mathbf{x}_{\mathrm{c}}(t) = e^{\mathbf{C}t}\int_0^t e^{-\mathbf{C}\tau}\mathbf{A}e^{\mathbf{S}\tau}\boldsymbol{\theta}\,d\tau$$

Returning to our R_0 formula:

$$R_0 = \frac{\boldsymbol{\beta}^{\mathrm{T}}\left(\int_0^\infty \left(1-F(t)\right)e^{\mathbf{C}t}\int_0^t e^{-\mathbf{C}\tau}\mathbf{A}e^{\mathbf{S}\tau}\boldsymbol{\theta}\,d\tau\,dt\right)}{\mathbf{1}^{\mathrm{T}}\int_0^\infty \left(1-F(t)\right)e^{\mathbf{S}t}\boldsymbol{\theta}\,dt}$$

We can substitute in $\mathbf{x}_{\mathrm{s}}$ and $\mathbf{x}_{\mathrm{c}}$ to arrive at an alternate, equivalent expression:

$$R_0 = \frac{\boldsymbol{\beta}^{\mathrm{T}} \int_0^\infty \big(1 - F(t)\big)\mathbf{x}_{\mathrm{c}}(t)dt}{\mathbf{1}^{\mathrm{T}} \int_0^\infty \big(1 - F(t)\big)\mathbf{x}_{\mathrm{s}}(t)dt}$$

We can numerically calculate the numerator and denominator of that expression using the following procedure:

Numerically solve for the eigenvalues and eigenvectors of the matrix $\mathbf{M}$. Place the eigenvalues in the diagonal matrix $\boldsymbol{\Lambda}$ and the eigenvectors in the corresponding columns of the matrix $\mathbf{V}$, then calculate:

$$\bar{\mathbf{x}} = \mathbf{V}K(\boldsymbol{\Lambda})\mathbf{V}^{-1}\boldsymbol{\theta}_{\mathbf{M}}$$

Then the integral $\int_0^\infty \big(1 - F(t)\big)\mathbf{x}_{\mathrm{s}}(t)dt$ consists of elements 1 through n of $\bar{\mathbf{x}}$, and $\int_0^\infty \big(1 - F(t)\big)\mathbf{x}_{\mathrm{c}}(t)dt$ consists of elements n through $n + m$ of $\bar{\mathbf{x}}$.

The product $\mathbf{V}^{-1}\boldsymbol{\theta}_{\mathbf{M}}$ is calculated by solving the system $\mathbf{Vx} = \boldsymbol{\theta}_{\mathbf{M}}$ for $\mathbf{x}$, which does not require calculating $\mathbf{V}^{-1}$.

If the matrix $\mathbf{M}$ has repeated eigenvalues, the above procedure fails when there is an incomplete set of linearly independent eigenvectors with which to construct a nonsingular matrix $\mathbf{V}$. In this case, our code attempts to calculate generalized eigenvectors to replace the duplicated eigenvectors in the matrix $\mathbf{V}$. If $\mathbf{v}$ is an eigenvector of $\mathbf{M}$ associated with a repeated, degenerate eigenvalue λ, then a generalized eigenvector $\mathbf{g}$ solves the equation $(\mathbf{M} - \lambda\mathbf{I})\mathbf{g} = \mathbf{v}$.

To calculate a solution for $\mathbf{g}$ numerically, we first compute the Moore-Penrose inverse, also known as the pseudoinverse, $(\mathbf{M} - \lambda\mathbf{I})^{+}$, of the matrix $(\mathbf{M} - \lambda\mathbf{I})$. The Moore-Penrose inverse $\mathbf{B}^{+}$ of a matrix $\mathbf{B}$ satisfies $\mathbf{BB}^{+}\mathbf{B} = \mathbf{B}$. It can be shown that $\mathbf{g} = \mathbf{B}^{+}\mathbf{v}$ is a solution to $\mathbf{Bg} = \mathbf{v}$. If we start with the equation to be solved and multiply both sides on the left by $\mathbf{BB}^{+}$, we get $\mathbf{BB}^{+}\mathbf{Bg} = \mathbf{BB}^{+}\mathbf{v}$. Using the definitional property of $\mathbf{B}^{+}$, the left-hand side is equivalent to $\mathbf{Bg}$, so $\mathbf{Bg} = \mathbf{B}(\mathbf{B}^{+}\mathbf{v})$, which shows that $\mathbf{g} = \mathbf{B}^{+}\mathbf{v}$ solves the equation.

We use a numerical method for the singular value decomposition of the matrix $(\mathbf{M} - \lambda\mathbf{I})$ to calculate $(\mathbf{M} - \lambda\mathbf{I})^{+}$ and then calculate a generalized eigenvector $\mathbf{g} = (\mathbf{M} - \lambda\mathbf{I})^{+}\mathbf{v}$. Then, we construct a Jordan matrix $\mathbf{J}$ to replace $\boldsymbol{\Lambda}$ and a matrix $\mathbf{P}$ containing eigenvectors and generalized eigenvectors such that $\mathbf{M} = \mathbf{PJP}^{-1}$. Then, the solution $\bar{\mathbf{x}} = \mathbf{P}K(\mathbf{J})\mathbf{P}^{-1}\boldsymbol{\theta}_{\mathbf{M}}$, and the component $K(\mathbf{J})$ includes elements above the diagonal involving derivatives of the function K evaluated at the repeated eigenvalue(s).

Facility outbreak transient dynamic simulation model

In the model described in the main text,

$$\frac{d\mathbf{x}}{dt} = \mathbf{Wx} - h(t)\mathbf{x}, \qquad \mathbf{x}(0) = \mathbf{x}_{\mathrm{a}},$$

the dependent variables $\mathbf{x}$ are the probabilities of a patient being in a given state as a function of the independent variable, t, which is the time since being admitted to the facility. We have shown that this form of the model can be used to generate both R_0 and the equilibrium distribution of patient states in the facility when the admission rate is constant and state-distributed according to $\mathbf{x}_{\mathrm{a}}$. However, this model does not describe the transient dynamics of patient states in a healthcare facility over time when they are not at equilibrium.

Transient facility dynamics could be modeled as follows. We redefine $\mathbf{x}(\tau, t)$ to represent facility patients in each state who were admitted τ time units ago, at time t after the start of the dynamic simulation. Then, the dynamic model can be written as a partial differential equation:

$$\frac{\partial\mathbf{x}}{\partial t} + \frac{\partial\mathbf{x}}{\partial\tau} = \mathbf{Wx} - h(\tau)\mathbf{x}, \qquad \mathbf{x}(0, t) = q\mathbf{x}_{\mathrm{a}}, \qquad \mathbf{x}(\tau, 0) = \mathbf{x}_0(\tau)$$

The differential on the left-hand side reflects that simulation time t and time-since-admission τ increase with each other in the same time units, so rates of state changes occur as a directional derivative along lines of slope 1 in the (τ, t) plane. The admission state distribution $\mathbf{x}_{\mathrm{a}}$ now appears as a boundary condition (at $\tau = 0$) and is multiplied by

a constant admission rate q. The function $\mathbf{x}_0(\tau)$ defines an initial condition (at $t = 0$) that describes the states and time-since-admission of patients who were admitted prior to simulation time 0 and have not yet been removed from the facility.

For models with $h(\tau) = 0$, i.e. when the facility patient removal rate is dependent only on the state of the patient and not explicitly on time-since-admission, the above model can be reduced to an ordinary differential equation for the state distribution, $\mathbf{X}(t) = \int_0^\infty \mathbf{x}(\tau, t)d\tau$, the states of facility patients at any time of their stay. We obtain the ordinary differential equation by integrating both sides of the partial differential equation:

$$\frac{\partial \mathbf{x}}{\partial t} + \frac{\partial \mathbf{x}}{\partial \tau} = \mathbf{W}\mathbf{x}$$

$$\int_0^\infty \frac{\partial \mathbf{x}}{\partial t} d\tau + \int_0^\infty \frac{\partial \mathbf{x}}{\partial \tau} d\tau = \int_0^\infty \mathbf{W}\mathbf{x}\, d\tau$$

$$\frac{d\mathbf{X}}{dt} + \big(\mathbf{x}(\infty, t) - \mathbf{x}(0, t)\big) = \mathbf{W}\mathbf{X}$$

$$\frac{d\mathbf{X}}{dt} + (0 - q\mathbf{x}_\mathrm{a}) = \mathbf{W}\mathbf{X}$$

$$\frac{d\mathbf{X}}{dt} = q\mathbf{x}_\mathrm{a} + \mathbf{W}\mathbf{X}, \qquad \mathbf{X}(0) = \int_0^\infty \mathbf{x}_0(\tau)d\tau = \mathbf{X}_0$$

When there is transmission occurring and the state distribution is not at equilibrium, the acquisition terms within $\mathbf{W}$ are non-constant because of the non-linear dependence on the number of patients in colonized, transmitting states changing with time. For example, for Model 1 from the main text we have the system:

$$\frac{\partial \mathbf{x}}{\partial t} + \frac{\partial \mathbf{x}}{\partial \tau} = \begin{pmatrix} -\beta C(t) & 0 \\ \beta C(t) & 0 \end{pmatrix} \mathbf{x} - h(\tau)\mathbf{x},$$

<u>where</u>

$$\mathbf{x}(\tau, t) = \begin{pmatrix} s(\tau, t) \\ c(\tau, t) \end{pmatrix}, \qquad C(t) = \int_0^\infty c(\tau, t)d\tau$$
